# Perineuronal nets restrict transport near the neuron surface: A coarse-grained molecular dynamics study

**DOI:** 10.3389/fncom.2022.967735

**Published:** 2022-11-17

**Authors:** Kine Ødegård Hanssen, Anders Malthe-Sørenssen

**Affiliations:** Department of Physics, University of Oslo, Oslo, Norway

**Keywords:** perineuronal net, molecular dynamics, coarse-grained molecular dynamics, polymer brush, diffusion, transport, resistance

## Abstract

Perineuronal nets (PNNs) are mesh-like extracellular matrix structures that wrap around certain neurons in the central nervous system. They are hypothesized to stabilize memories in the brain and act as a barrier between cell and extracellular space. As a means to study the impact of PNNs on diffusion, the nets were approximated by negatively charged polymer brushes and simulated by coarse-grained molecular dynamics. Diffusion constants of single neutral and single charged particles were obtained in directions parallel and perpendicular to the brush substrate. The results for the neutral particle were compared to different theories of diffusion in a heuristic manner. Diffusion was found to be considerably reduced for brush spacings smaller than 10 nm, with a pronounced anisotropy for dense brushes. The exact dynamics of the chains was found to have a negligible impact on particle diffusion. The resistance of the brush proved small compared to typical values of the membrane resistance of a neuron, indicating that PNNs likely contribute little to the total resistance of an enwrapped neuron.

## 1. Introduction

Perineuronal nets (PNNs) are mesh-like extracellular matrix structures that ensheath the soma and proximal dendrites of neurons in the brain, particularly parvalbumin-positive (PV) interneurons (van 't Spijker and Kwok, 2017). They have garnered particular interest over the last two decades because of their proposed role in stabilizing long-term memory (Pizzorusso et al., 2002; Tsien, 2013), a hypothesis which have later been corroborated by experiments (Thompson et al., 2018; Carulli et al., 2020). The nets have a complex structure and consist to a large degree of glycosaminoglycans, a family of long, unbranched and highly negatively charged polysaccharides. Due to their large negative charge, the nets are hypothesized to restrict ion transport (van 't Spijker and Kwok, 2017). Developing an improved understanding of how PNNs can act as transport barriers is important because ion concentration and transport affect cell function.

PNNs may be considered to comprise a porous system, with similarities to polymer brushes. However, the exact structure of PNNs remains difficult to characterize. The impact of the structure of PNNs on transport into, out of and around neurons is therefore sparsely understood. Given the limited insight into the precise structure of PNNs, it is important to develop an improved understanding of the general properties of diffusional transport in polymer networks and brushes that reflect general aspects of PNNs. While the fundamental process of diffusion has been well understood since Einstein's seminal paper in 1905 (Einstein, 1905; Tartakovsky and Dentz, 2019), our understanding of diffusion near surfaces and inside tight porous media is still being developed. For example, recent studies address surface diffusion (Postl et al., 2022), diffusion inside nanotubes (Zelenovskiy et al., 2020) and inside planar polymer brushes (Wetzler et al., 2020; Luo et al., 2021) or diffusion of spherical brushes in a polymer melt (Chen et al., 2022). Diffusion in polymer brushes are also of interest in other applications, such as for our understanding of lubricants (Spirin et al., 2011) and for nanoscience and technology (Zhang and Xiang, 2013).

Computational studies of diffusional transport can complement experimental studies in particular in cases where detailed measurements prove difficult, like inside shearing brushes (Ou et al., 2012). Molecular dynamics (MD) simulations have the advantage of yielding deterministic, time-dependent trajectories. A number of molecular dynamics studies have been conducted both on polymers in general and polymer brushes in particular. Singh et al. (2015) applied implicit solvent molecular dynamics simulations through the Langevin equation on apposing neutral polymer brushes represented by a coarse-grained bead-spring model without bending terms in the potential. The density profiles, compression curves and friction was found. Studies of charged apposing brushes using implicit solvent were performed by Ou et al. (2012) and Cao et al. (2010). Both papers found the friction coefficient μ, monomer density profile ρ_*m*_ and degree of interpenetration *I* under shear motion for two apposing charged polymer brushes with bond stiffness and steric effects through the finitely extensible linear elastic (FENE) (Kremer and Grest, 1990) and Lennard-Jones (LJ) potentials, but no potential accounting for bending stiffness. The osmotic pressure and virial terms were plotted against the separation distance by Cao et al. (2010). Ou et al. (2012) found the normal pressure for three different separation distances, but put effort into comparing μ, ρ_*m*_ and *I* of apposing charged and apposing neutral brushes with different counterion valency. Singh et al. (2016) studied the effect of chain stiffness, grafting density and wall separation on apposing electrically neutral polymer brushes by incorporating a tunable cosine bending potential, finding the normal stress, shear stress and friction coefficient. Additionally, they probed the structure by measuring the brush height and number density as a function of chain stiffness and grafting density. A relation between the radius of gyration *R*_*g*_ and the of bending stiffness was found. Unlike the papers of Cao et al. (2010), Ou et al. (2012), Singh et al. (2015), and Singh et al. (2016) made use of explicit water.

MD studies of insertion forces, inclusion free energy and osmotic pressure in coarse-grained polymer brushes were performed for particles of different shapes and sizes by de Beer et al. (2016) and different sizes, solvent conditions and degree of polydispersity by Merlitz et al. (2012) using the LJ and FENE potential and implicit solvent through the Langevin equation.

Brush configurations were studied by Li et al. (2018) who investigated the effect of counterion valence on the configuration of a spherical brush confined between two planes. The structure was probed by the end monomer distribution, ρ_*m*_, brush thickness, *R*_*g*_, and the radial distribution function *g*_*np*_ between brush monomers and counterions. Lastly, the mean square displacement of the counterions was found in directions parallel and perpendicular to the confining planes. The LJ, FENE and Coulomb potentials were applied.

Zhang and Xiang (2013) studied diffusion of a single free nanoparticle in a polymer brush through the particle-wall distance *z*_*np*_, ρ_*m*_, the lateral diffusion coefficient *D*_∥_ and the force on the particle *F*_*np*_. They found a decrease of *D*_∥_ and increase of *z*_*np*_ with increasing grafting density. The simulation was set up using the Langevin equation and the LJ and FENE potentials. Csajka and Seidel (2000) used the same interactions, but also included the Coulomb potential in order to study polyelectrolyte brushes using MD. Instead of one diffusing particle, they had several counterions inside the brush. Their analysis included the counterion diffusion constants parallel and perpendicular to the plane, *D*_*xy*_ and *D*_*z*_, which were both found to increase with the grafting density due to a decrease in the number of condensed counterions for denser brushes. While the work of Csajka and Seidel provides broad insight into polyelectrolyte brushes, it does not include a potential accounting for bending stiffness. Due to the semi-flexible nature of the constituents of the perineuronal nets (Richter et al., 2018), additional simulations are therefore required in order to understand diffusion in the PNNs. Furthermore, neither (Zhang and Xiang, 2013) nor (Csajka and Seidel, 2000) attempted to compare the diffusion constant to existing models of diffusion. Important insights might be gained from such models. Finally, estimating the effective resistance due to ion diffusion through a model polymer brush system may provide insights into the effects of PNNs on nerve signal propagation.

In this paper, we study diffusion in a charged, coarse-grained planar polymer brush by use of molecular dynamics as a first attempt at a model for diffusion in PNNs. The root mean square displacement of a single neutral or a single charged particle obtained through 1,000 system realizations is utilized to obtain the diffusion coefficient in directions perpendicular and parallel to the explicit substrate plane. Lennard-Jones, harmonic stretching and harmonic bending potentials are included in order to account for steric interactions, the stiffness of the bond and chain flexibility. Debye interactions account for screened electrostatic forces due water polarizability and dissolved ions in the liquid. The diffusion constants are found as a function of polymer chain spacing and compared to theory for a neutral particle. The diffusion constant for particles of various charges are then obtained and used to estimate the resistance of a 500 nm thick brush.

## 2. Model

**Figure 1:**
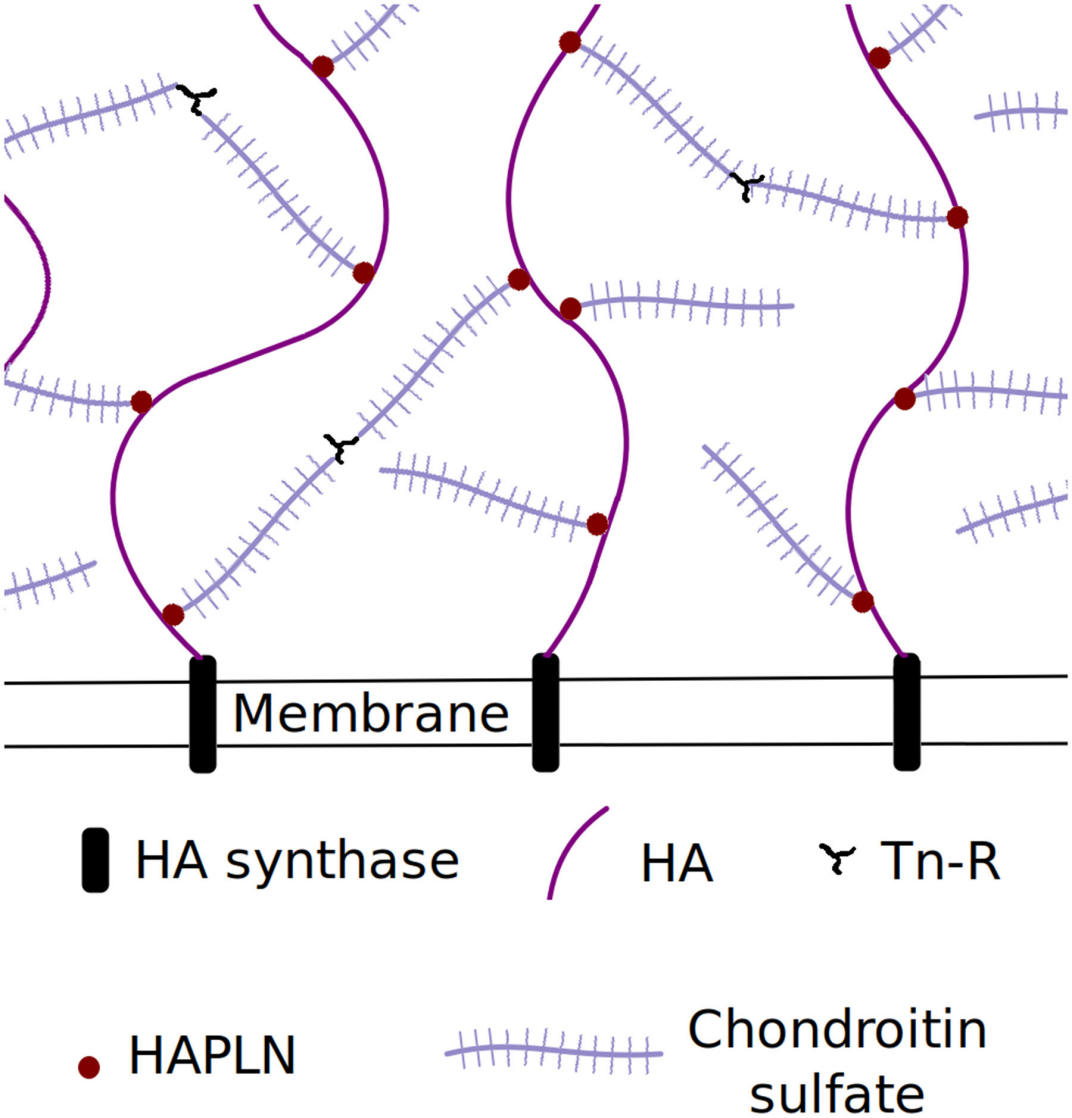
A schematic of the perineuronal net structure.

Perineuronal nets consist of hyaluronan, chondroitin sulfate proteoglycans (CSPGs), Tenascin-R (Tn-R) and HAPLNs, the first acting as a scaffold and the latter two being cross-linkers (Richter et al., 2018). Hyaluronan (HA) and chondroitin sulfate (CS) are glycosaminoglycans (GAGs), which are unbranched, highly negatively charged sugar molecules of significant length (Richter et al., 2018). Although detailed information on the PNN structure is not known, it is hypothesized to look like Figure 1 (Richter et al., 2018). The nets form a cross-linked brush that is locally planar due to the relative sizes of the nets and the cell. As visible from Figure 1, several length scales are present in the system, namely the length of the chondroitin sulfate chains on the CSPGs, the length of the protein backbone of the CSPGs, the distance between chondroitin sulfate chains on the CSPGs, the distance between CSPGs on the hyaluronan chains, the length of the hyaluronan and the distance between neighboring hyaluronan chains. While a detailed model would be ideal, few of these PNN parameters are known. Instead of implementing a complicated, large-scale structure for which the exact structure is not known, the system was simplified to a planar glycosaminoglycan brush without attached CSPGs and cross-links in order to study diffusion in a GAG-rich structure. Investigating the effect of cross-linking and side-chains is left to future works.

**Figure 2:**
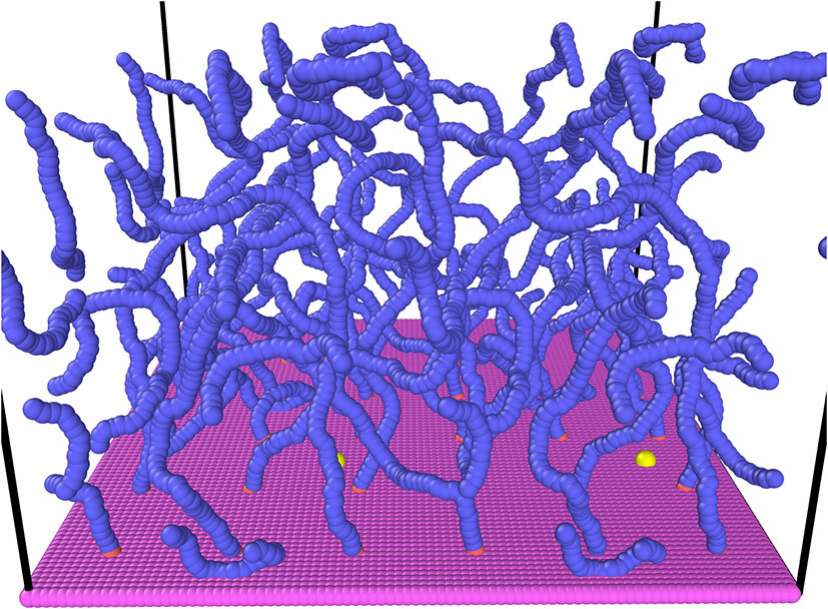
Snapshot of the modeled system for *d* = 15σ. The snapshot is rendered in ovito with two periodic images in the *x*- and *y*- directions. Magenta beads-wall; coral beads-the two first beads of the chain (kept in place to ensure tethering to wall); blue beads-chain elements; yellow bead-diffusing particle (slightly enlarged for visibility). There is more than one yellow bead visible due to the periodic images, while only one particle is diffusing in the simulations.

As glycosaminoglycans are made up of repeating disaccharide units (Richter et al., 2018), larger systems can be modeled by coarse-graining the disaccharides into one bead instead of having explicit particles for every atom. One bead has length 1 nm, corresponding to a disaccharide unit of HA or CS, and the charge is −*e* just like in HA or unsulfated chondroitin. The disaccharide beads are linked together to form chains of *N* = 101 beads each, tethered to the membrane through two immobile beads. This length is short for membrane-attached HA, but a compromise was made in order to study sufficiently large system sizes. The chains proved sufficiently long to observe the most important effects of PNNs. The immobile beads form a 3 × 3 quadratic grid with lattice spacing *d*. To adhere to the GAG structure, chains are unbranched. Chains are kept flexible and of appropriate length through adjustable bond stretching and bond bending interactions, while the Lennard-Jones interaction prevents overlaps. The membrane is modeled by a rectangular lattice of immobile beads, whose LJ potential prevents other beads from passing through. A single unbonded particle is inserted close to the wall to probe diffusion in the brush. Periodic boundary conditions are applied in directions parallel to the substrate. A snapshot of the system is provided in Figure 2. The snapshot is rendered in ovito (Stukowski, 2009), and two periodic images are included.

## 3. Methods

### 3.1. Simulation setup

In order to capture properties of diffusion in the PNNs, molecular dynamics simulations of one particle in a system of tethered negatively charged polymer chains on a substrate were performed. The lammps molecular dynamics package (Thompson et al., 2022) was used to run the simulations. Simulations were performed for 1,000 realizations of the system in order to gain appropriate statistics. Each realization had a different starting configuration of the particle and the chain beads.

The potentials used in the molecular dynamics simulations were selected to capture key properties of the PNNs. The Lennard-Jones potential was used to account for the spatial extent of the molecules. We have chosen the truncated 12–6 Lennard-Jones potential as it is common in the literature (see e.g., Merlitz et al., 2012). The potential $U_{\text{LJ}}=\underset{i<j}{\sum }u_{\text{LJ}}\left(r_{ij}\right)$ works between all pairs of beads, including wall beads


(1) $$\begin{array}{l} u_{\text{LJ}}\left(r\right)=4\epsilon_{\text{LJ}}\left[{\left(\frac{\sigma_{\text{LJ}}}{r}\right)}^{12}-{\left(\frac{\sigma_{\text{LJ}}}{r}\right)}^{6}\right],\;r<r_{c,\text{LJ}}, \end{array}$$

where ϵ_LJ_ is the interaction strength and *r* is the distance between the beads. σ_LJ_ is the Lennard-Jones distance, which is related to the separation distance *r*_min_ of minimal potential energy by $r_{\text{min}}=2^{1/6}\sigma_{\text{LJ}}$. *r*_*c*, LJ_ is the cutoff for the Lennard-Jones force.

As the repeating units of the GAGs have an approximately fixed size, we introduce a stretching potential following e.g., Zhang and Xiang (2013) and Csajka and Seidel (2000). Preliminary simulations showed that similar bond lengths were obtained using both the FENE potential (Kremer and Grest, 1990) and a harmonic stretching potential. We therefore chose a harmonic stretching potential as for example done by Horkay et al. (2020) and Kinjo et al. (2018). The harmonic stretching potential $U_{\text{bond}}=\underset{i<j}{\sum }u_{\text{bond}}\left(r_{ij}\right)$ acts between every bonded pair of beads and is given as


(2) $$\begin{array}{l} u_{\text{bond}}\left(r\right)=K_{\text{bond}}{\left(r-r_{0}\right)}^{2}, \end{array}$$

where *K*_bond_ is the interaction strength, *r*_0_ is the equilibrium bond length, and *r* is the distance between the bonded neighbors.

As the glycosaminoglycans comprising the majority of the PNNs are semi-flexible with a well defined persistence length (Richter et al., 2018), a harmonic bond bending potential was introduced as in Hehmeyer and Stevens (2005). The potential $U_{\text{bend}}=\underset{i<j<k}{\sum }u_{\text{bend}}\left(\theta_{ijk}\right)$ acts between every subsequent pair, *ij*, *jk*, of neighboring bonds


(3) $$\begin{array}{l} u_{\text{bend}}\left(\theta \right)=K_{\text{bend}}{\left(\theta -\theta_{0}\right)}^{2}, \end{array}$$

where *K*_bend_ is the interaction strength, θ_0_ is the equilibrium angle, and θ is the angle between two subsequent bonds.

As the PNNs possess a highly negative charge, electrostatic interactions also need to be included. Furthermore, the cerebrospinal fluid contains dissolved ions that will screen charges in the extracellular matrix. The electrostatic interaction was therefore represented by the Debye potential. The Debye potential $U_{\text{Debye}}=\underset{i<j}{\sum }u_{\text{Debye}}\left(r_{ij}\right)$ acts between all pairs of charged beads


(4) $$\begin{array}{l} u_{\text{Debye}}\left(r\right)=\frac{Cq_{i}q_{j}}{\epsilon r}e^{-\kappa r},\;r<r_{c,\text{Debye}}, \end{array}$$

where *q*_*i*_ and *q*_*j*_ are the bead charges, κ is the inverse of the Debye length (Nelson, 2014) and *r*_*c*, Debye_ is the cutoff for the interaction. *C* is an energy-conversion constant and ϵ is the dielectric permittivity. We assume that the Debye potential approximation is reasonable for modeling diffusion in interstitial fluids in the brain, with a Debye constant of approximately 1 nm (Syková and Nicholson, 2008).

The potential energy of the system is given by the sum of the stretching potential, the bending potential, the Lennard-Jones potential and the Debye potential


(5) $$\begin{array}{l} U=U_{\text{bond}}+U_{\text{bend}}+U_{\text{LJ}}+U_{\text{Debye}}. \end{array}$$

As incorporating explicit water molecules and ions is computationally expensive, the solvent was modeled implicitly. The Langevin equation is utilized as it mimics interactions with water by adding terms representing random collisions and drag effects of the fluid:


(6) $$\begin{array}{l} m\frac{d\text{v}}{dt}=\text{F}\left(t\right)-\gamma \text{v}+\text{B}\left(t\right), \end{array}$$

where **B**(*t*) is a random term (Lemons and Gythiel, 1997). The damping coefficient γ is defined as γ = 6π*aη* where *a* is the radius of the particle and η is the liquid viscosity.

Simulations were run for several types of systems. For the dynamic brush system (abbreviated as Dyn.), both the chains and the diffusing particle were considered dynamic particles and their time dynamics were calculated using molecular dynamics. For the static brush system (Stat.), the chains were modeled as static configurations by first equilibrating the chains, then fixing the chains while allowing the free particle to diffuse. This allowed us to compare the static and the dynamic system to provide insight into the role of geometry and flexibility on the diffusional properties of the system. In addition, simulations were performed on a dynamic brush system without bending terms (No stiff.) and in a static system with completely straight chains (Straight). Interactions between all particles, static or dynamic, were included in force and energy calculations.

Two types of grid geometries of the chain tethering points were studied: a quadratic and a hexagonal grid. However, preliminary simulations showed no systematic difference in the measured properties for the two grid types as illustrated in Supplementary Figure S1. We therefore limited the study to quadratic grids only.

A 3 × 3 quadratic grid was deemed sufficient as periodic boundary conditions were applied and no long-range forces were present. To verify that this assumption holds, the diffusion constants were found for systems of size 3 x 3, 4 x 4, 5 x 5, 6 x 6, and 9 x 9 for spacing *d* = 3σ. The diffusion constants agreed within the standard deviation. Furthermore, the sheer number of realizations for each grid spacing *d* should ensure that spatial heterogeneities are taken into account.

#### 3.1.1. Parameter selection

The persistence length of a polymer characterizes its bending stiffness and is defined as the separation distance for which the correlation of bond vectors have fallen by 1/*e*. Its mathematical expression may be given as follows Kamerlin (2017):


(7) $$\begin{array}{l} \langle \cos\theta \left(s\right)\rangle =e^{-s/l_{p}}, \end{array}$$

where θ(*s*) is the angle between two bonds separated by the distance *s* along the polymer and *l*_*p*_ is the persistence length. For distances smaller than *l*_*p*_, the bond orientations display a significant correlation (Kamerlin, 2017).

The parameters of the polymer model were tuned to yield a persistence length of 10 nm in accordance with Bathe et al. (2005), resulting in interaction parameters of *K*_bond_ = 140.2 and *K*_bend_ = 14.02. Lennard-Jones units were used, and the unit length σ was set to the length of a disaccharide unit, that is the bond length, so that σ = *r*_0_ = 1 nm (Richter et al., 2018). The mass of the disaccharide unit is set to unity. The equilibrium angle was set to θ_0_ = π in order to recreate the relatively linear nature of glycosaminoglycans.

In the Lennard-Jones interaction, the cutoff *r*_*c*, LJ_ is set to 1.122σ_LJ_ to coincide with the energy minimum at $r_{\text{min}}=2^{1/6}\sigma_{\text{LJ}}$, corresponding to good solvent conditions. The Lennard-Jones distance σ_LJ_ should be sufficiently large to avoid overlap between monomers. This is accomplished by ensuring that σ_LJ_ is equal to the length of a disaccharide unit, which means that σ_LJ_ = σ for chain beads. In the simulations the diffusing particle is the same size as the chain beads, σ_LJ_ = σ, except when finding *D*_bulk_ as a function of the particle radius *a* = σ_LJ_/2. The interaction parameter ϵ_LJ_ = 0.73 in LJ units is derived from the carbon-carbon interaction parameter of 0.15 kj/mol that (Bathe et al., 2005) used in their coarse-graining of GAGs.

The chain beads hold a charge of −*e* each, corresponding to the charge of the repeating disaccharide unit of HA and unsulfated chondroitin (Richter et al., 2018). The substrate is neutral while the diffusing particle may be charged or neutral. The Debye length was set to match that of the brain extracellular fluid, namely 1/κ = σ = 1 nm (Syková and Nicholson, 2008), and the dielectric permittivity was set to match that of water. A cutoff of $r_{c,\text{Debye}}=3\kappa^{-1}=3\sigma$ was implemented in agreement with Bathe et al. (2005).

## 4. Results

### 4.1. Anisotropic diffusion

A well-known result in physics is that the diffusion constant *D*_*R*_ in an isotropic system is related to the mean square distance 〈Δ*R*^2^(*t*)〉 traveled by the diffusing particles. In three dimensions, it takes the mathematical form of


(8) $$\begin{array}{l} \langle \Delta R^{2}\left(t\right)\rangle =\langle \Delta x^{2}\left(t\right)+\Delta y^{2}\left(t\right)+\Delta z^{2}\left(t\right)\rangle =6D_{R}t, \end{array}$$

where Δ*x*^2^(*t*), Δ*y*^2^(*t*) and Δ*z*^2^(*t*) are the squared displacements in the *x*-, *y*- and *z* directions at time *t*, and Δ*R*^2^(*t*) is the total displacement.

The presence of a wall limits transverse motion, resulting in an anisotropic diffusion constant. Anisotropic diffusion has also been observed in brain areas with high concentration of PNNs (Morawski et al., 2015). In order accurately characterize diffusion in such systems, the mean square distance needs to be separated into contributions in directions parallel and perpendicular to the wall, as done by Carbajal-Tinoco et al. (2007)


(9) $$\begin{array}{l} \langle \Delta x^{2}\left(t\right)+\Delta y^{2}\left(t\right)\rangle =4D_{∥}t \end{array}$$

(10) $$\begin{array}{l} \langle \Delta z^{2}\left(t\right)\rangle =2D_{⊥}t. \end{array}$$

Here, *D*_∥_ and *D*_⊥_ are the diffusion constants in directions parallel (*xy*-plane) and perpendicular (*z*-axis) to the wall, respectively. Note that Equations (9) and (10) are valid in the diffusive regime, i.e., only for time scales longer than the initial ballistic regime. Strictly speaking, the diffusion constant of the particle should be a function of the particle-wall distance due to the anisotropy posed by the substrate. However, since the overall diffusive properties inside the nets are of interest, this is considered a detail outside the scope of this paper.

To ensure that the diffusion constant is characteristic for the brush, two versions of the mean square displacement (MSD) curve were found. The first curve is the MSD for the particle at all times regardless of whether or not it is still in the brush. At each point in time, the second curve is the MSD of particles still inside the brush. We disregard the initial part of the graphs, where inertial effects are prominent and the MSD curves are non-linear. The fits to Equations (9) and (10) were performed on an interval where the two MSD curves had similar slopes. In order to obtain error estimates, the MSD curves were divided into groups of 100 so that ten estimates of *D*_∥_ and *D*_⊥_ could be obtained using NumPy's polyfit function (Harris et al., 2020). The average and standard deviation were calculated from these ten values. A plot illustrating the procedure is given in Supplementary Figure S2.

The free particle is subject to interactions with chain- and substrate beads in the system. For interacting particles, two diffusive regimes occur. On shorter time scales, the particle moves close to a local energy minimum in the energy landscape (Dhont, 1996). As time progresses, the particle passes many energy barriers and visits many local minima. Additionally, changes in brush configuration alter the potential energy landscape, creating new minima for the particle to visit. The short-time and long-time diffusion constants are therefore different. Between these time scales, where only a few minima has been visited, the MSD is non-linear (Dhont, 1996). Our MSD plots did not display such a non-linear interval while the particle was inside the brush, indicating that long-time diffusion was not reached. Note that particles exiting the brush puts an upper limit on the time scale of diffusion.

**Figure 3:**
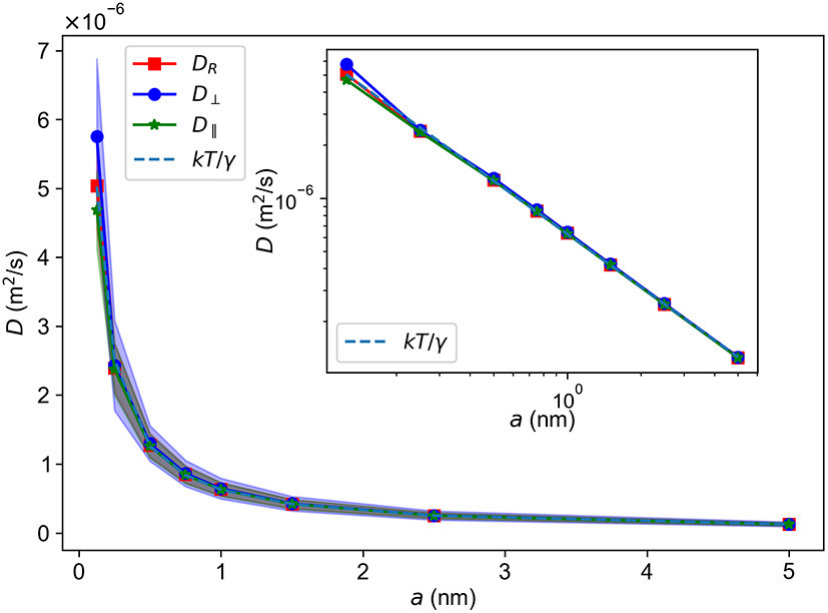
Diffusion constant *D* vs. radii *a* of a free particle in bulk. *D*_*R*_ - total diffusion constant; *D*_⊥_-diffusion constant in the *z*-direction; *D*_∥_-diffusion constant in the *xy*-plane; *kT*/γ-the theoretical value of *D*. Standard deviations are indicated by shaded regions. Insert: A log-log plot of the same data.

Figure 3 shows the bulk diffusion constant *D*_bulk_ as a function of the radius *a* of the diffusing particle found by Equations (8)–(10). The bulk diffusion constant was found by tracking the movement of a single particle subject to only Equation (6), where γ depends on *a*. The system is otherwise empty, meaning that **F**=0 in Equation (6) in this instance. We see a sharp decrease of *D*_bulk_ with increasing *a*. The diffusion constants in all directions are within the error margins of each other, as expected due to the isotropic nature of the bulk system. The insert in Figure 3 shows a log-log plot of *D*_bulk_ vs. *a*. The curves appear linear except for some small deviations at the lowest value of *a* = 0.125 nm. The data is in agreement with the Stokes-Einstein equation *D* = *kT*/(6π*ηa*) (indicated by the dashed line) within the standard deviation (Edward, 1970).

### 4.2. Brush configuration vs. spacing *d*

**Figure 4:**
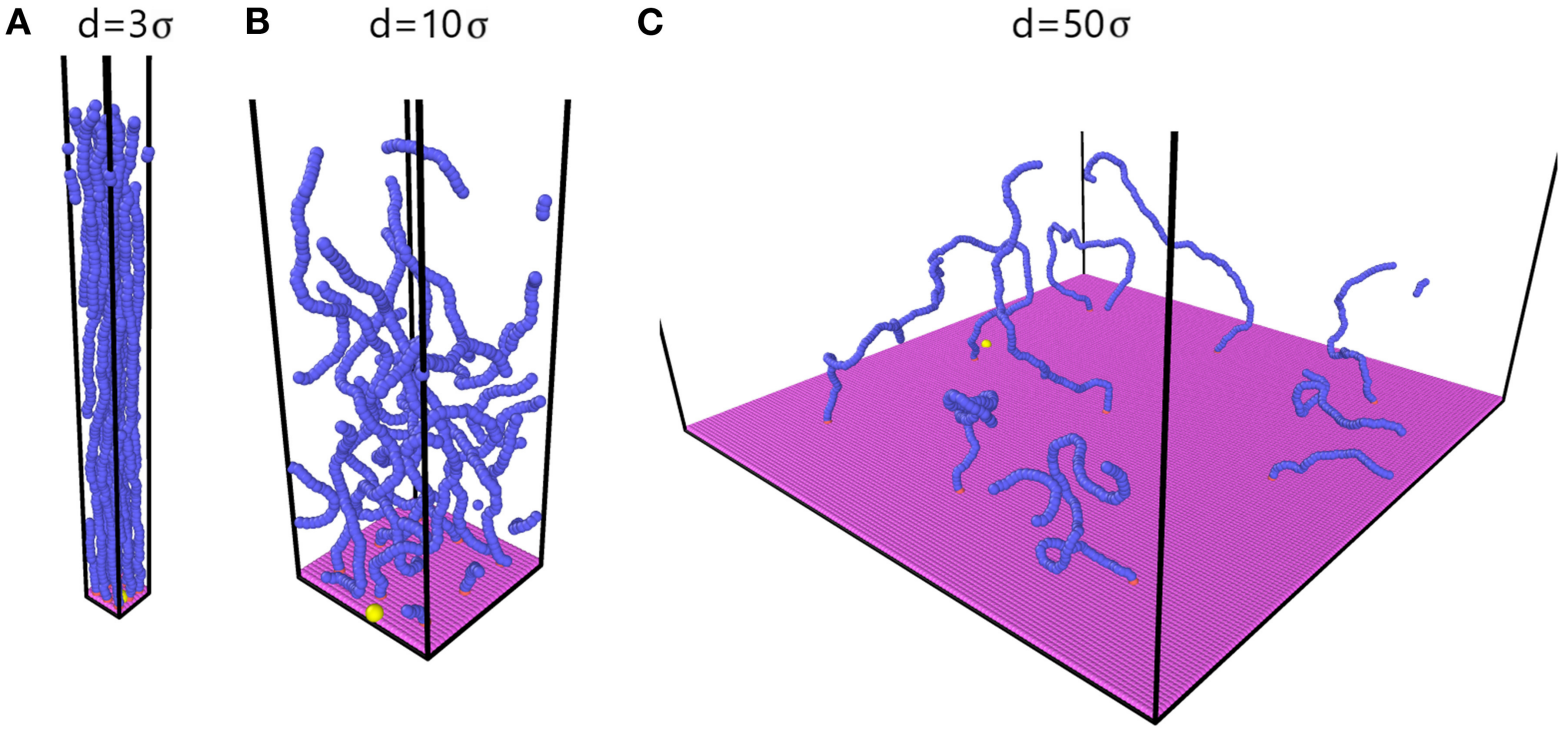
Brush configurations for different spacings *d*. **(A)** *d* = 3σ, **(B)** *d* = 10σ and **(C)** *d* = 50σ. The chains are much straighter in A than in C. Magenta beads-wall; blue and coral beads-chain beads; yellow bead-free particle (slightly enlarged for visibility). The figure is rendered in ovito.

Figure 4 shows system configurations for spacings of *d* = 3σ, *d* = 10σ and *d* = 50σ. For the smallest values of *d*, such as *d* = 3σ in Figure 4A, the chains are far straighter than for larger spacings such as *d* = 10σ and *d* = 50σ. Table 1 shows the potential energy of the neutral diffusing particle and reveals that at small *d*, the particle experiences both stronger and more frequent interactions with other beads in the system. Also included in Table 1 is the average potential energy of the chain beads. The chain beads experience similarly increased Debye and Lennard-Jones interactions at small *d*, forcing them into straighter configurations in order to lower the energy. For *d* < 2σ, visualization of the trajectories revealed an initial period of random motion until the diffusing particle starts moving back and forth within a small volume in the brush. A video of a trajectory for *d* = 1.5σ is provided in Supplementary Video S1. Keeping in mind that the Lennard-Jones interaction has a minimum at $2^{1/6}\sigma_{\text{LJ}}$ and that σ_LJ_ = σ for both particle and beads in these simulations, the particle will experience significant forces from multiple chains propelling it forwards until it happens to encounter a cavity within the brush. If the particle velocity is sufficiently small or the cavity is sufficiently large, the particle will not be able to move forward, but will be trapped inside the cavity. At longer time scales, the particle would typically have been able to escape this volume, displaying a reduced but non-zero diffusion constant in the long-time diffusion regime (Cai et al., 2011). Such a diffusion regime was not reached for our simulations.

**Table 1:** Potential energy of the free particle and the brush.

***d* (σ)**	**1**	**3**	**10**	**50**
**Particle**				
∑*E*/*N*_*t*_ (ϵ_E)	0.0027 ± 0.0010	1.0e-4 ± 1.2e-4	8.0e-6 ± 3.4e-5	1.2e-7 ± 2.6e-7
*E* (ϵ_E)	0.0027 ± 0.0010	8.0e-4 ± 3.4e-3	5.0e-4 ± 3.4e-3	2.7e-5 ± 2.8e-4
*N*_*E*_	0.9997 ± 0.0005	0.179 ± 0.026	0.023 ± 0.010	0.007 ± 0.006
**Chain**				
∑*E*/*N*_*t*_ (ϵ_E)	281.0 ± 0.6	9.94 ± 0.25	2.14 ± 0.04	0.1634 ± 0.0016

Average potential energy ∑*E*/*N*_*t*_ for the diffusing particle along with the average size *E* of the potential energy *E* when *E* ≠ 0, and the fraction of time steps *N*_*E*_ with a non-zero potential energy *E* ≠ 0 for selected spacings *d*. Only ∑*E*/*N*_*t*_ is included for the potential energy of the chain beads as the chain beads are interacting with their neighbors and the substrate at all times. All energies are per atom. ϵ_E = 1.4*10^−21^ J.

As *d* increases and beads on different chains get farther apart, the magnitude of the interactions between beads on different chains decreases, as seen in Table 1, and the chains take on a more curled configuration, as seen for *d* = 10σ in Figure 4B. As *d* is increased further, interchain interactions will rarely occur. The system is then said to be in the mushroom configuration, which is typically distinguished from the brush configuration (Kim et al., 2015).

In summary, the system is shown to enter three regimes: The extremely dense brush for *d* < 2σ, in which neutral particles get trapped at our time scales, the mushroom regime for large *d*, in which chains are far apart and we expect diffusion to be similar to bulk diffusion, and the brush regime for intermediate values of *d*. Simulations for large values of *d* have been performed in order to verify that the system tends to bulk behavior in the limit of large *d*. Intermediate *d*-values are of primary interest, as they yield systems similar to that of perineuronal nets. We therefore select *d* = 2σ as the lower limit for *d*, ensuring that we study a range of *d*-values for which the particle is sufficiently mobile in most of the simulations. The aptness of this limit can be seen by comparing the mean square displacement in Supplementary Figures S3, S4.

**Figure 5:**
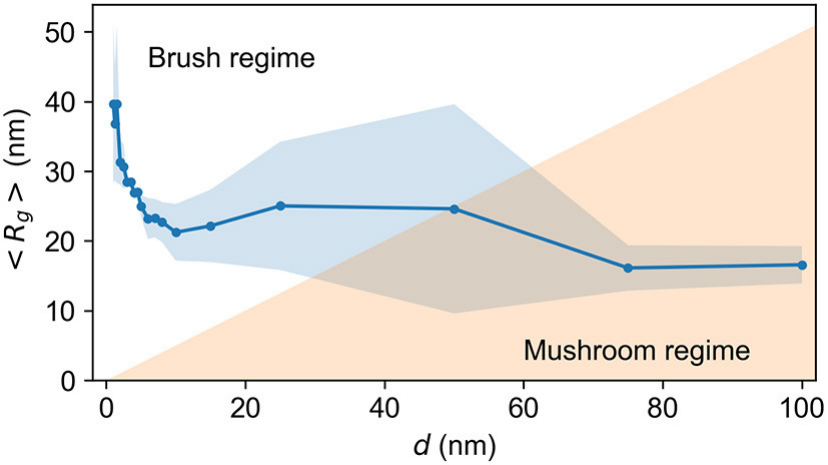
*R*_*g*_ vs. *d* for the dynamic brush. The grafted polymers are in the brush configuration for *d* < 2*R*_*g*_ and in the mushroom configuration for *d* > 2*R*_*g*_ (Kim et al., 2015), as indicated by the colored background. The standard deviation is indicated by the shaded blue area.

Tethered polymers are in the brush configuration for *d* < 2*R*_*g*_ (Kim et al., 2015), where *R*_*g*_ is the radius of gyration, which for a polymer chain is defined as Kamerlin (2017):


(11) $$\begin{array}{l} R_{g}=\sqrt{\frac{1}{N}\overset{N}{\underset{i=1}{\sum }}{\left({\text{r}}_{i}-\bar{\text{r}}\right)}^{2}}, \end{array}$$

where *N* is the number of monomers in a chain, **r**_*i*_ is the position of the *i*th monomer and $\bar{\text{r}}$ is the center of mass. Figure 5 shows 〈*R*_*g*_〉 vs. *d* for the dynamic brush plotted in the form of a phase diagram, where the brush and mushroom regimes are indicated by the background color. We found 〈*R*_*g*_〉 by averaging *R*_*g*_ in Equation (11) over all chains and realizations for the last time frame of each simulation. Based on Figure 5 we find that the system is in the brush configuration for *d* ∈ [2σ, 25σ], whereas for larger values of *d* the system is in the mushroom configuration. Here, we have focused on the brush regime which is most similar to PNN structure, and have primarily analyzed systems with *d* < 25σ.

To compare the brush configuration to theoretical predictions, the average height of the brush *L*_*z*_ was found for each *d* by extracting the maximal *z*-coordinate of the chain beads at the last time frame and averaging over all realizations. The brush height as calculated by mean field theory scales with brush spacing as $L_{z}=Ad^{-2/3}$ (Attili et al., 2012), where *A* is a constant that does not depend on *d*. The data points were fit to theory using the Levenberg-Marquardt algorithm as implemented in SciPy's optimize.curve_fit function (Virtanen et al., 2020). The height of the brush agreed fairly well with the theory, as shown in Supplementary Figure S5 and Supplementary Table S1, where fits to theory are included as dashed lines. The height of the brush without bending potential energy showed the best agreement with the theory, while the systems with bending stiffness also displayed an acceptable agreement. The system types are as defined in Section 3.1.

### 4.3. Diffusion constant *D* vs. grid spacing *d*

#### 4.3.1. Dynamic brush

**Figure 6:**
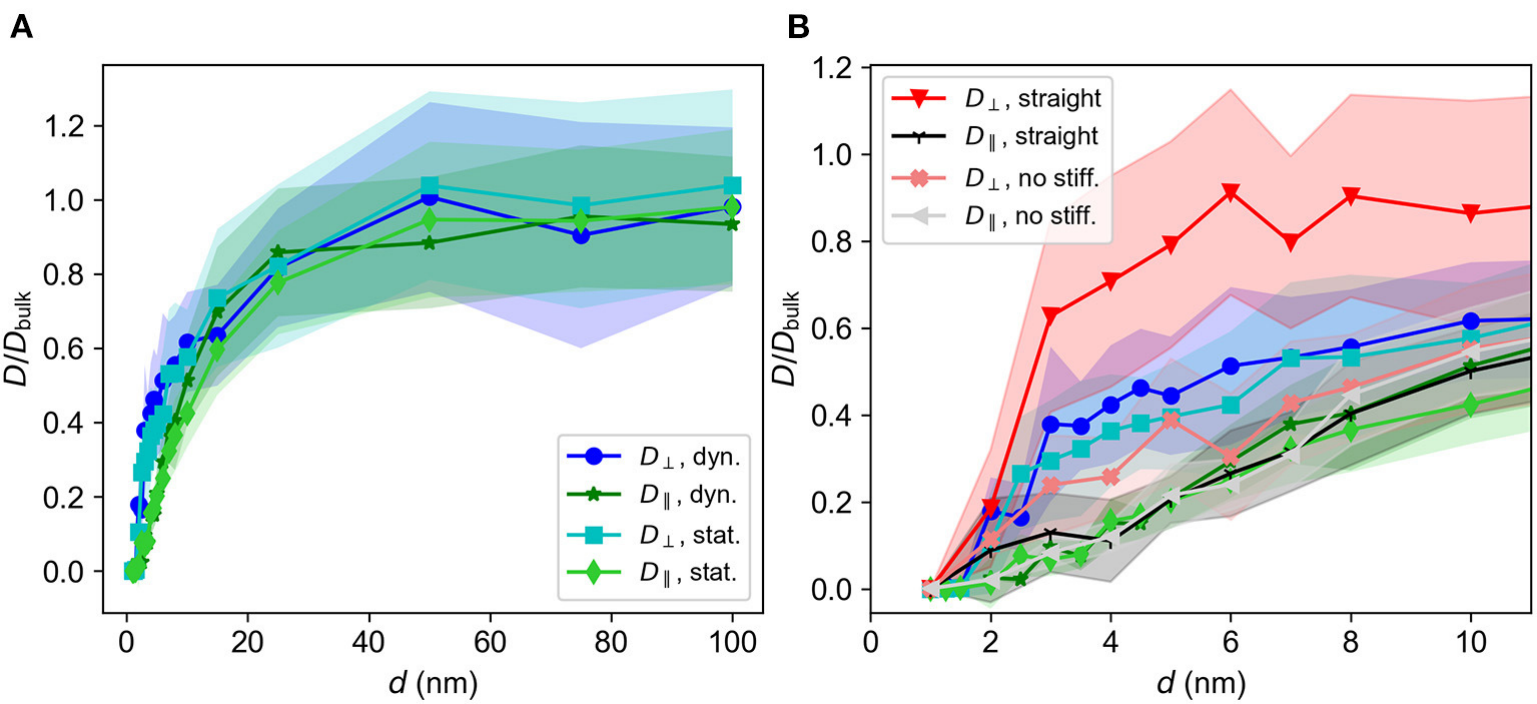
*D*/*D*_bulk_ vs. *d* in different directions for a neutral particle in dynamic and static brushes. **(A)** *D*/*D*_bulk_ for *d* ∈ [1σ, 100σ]. *D*/*D*_*bulk*_ ≈ 0 for *d* < 2σ, then increases with *d* and eventually converges to 1. **(B)** *D*/*D*_bulk_ for *d* ≤ 10σ. Diffusion constants for straight, immobile chains and chains without a bending term are included. Blue line, *D*_⊥_; green line, *D*_∥_. Bold colors, dynamic brush; light colors-static brush. Red, *D*_⊥_ for the straight system; light red-*D*_⊥_ for the system without bending stiffness. Black, *D*_∥_ for the straight system; light gray, *D*_∥_ for the system without bending stiffness. Standard deviations are indicated by shaded regions.

Figure 6A shows *D*(*d*)/*D*_bulk_ for the dynamic and the static brush. This ratio converges to unity when the lattice spacing *d* increases, which is as expected since the system effectively approaches bulk as *d* becomes large. The decreasing values of *D* as *d* becomes smaller are due to brush interactions, which become more dominant as the density of the brush increases. At *d* < 2σ frequent entrapment yields an effective diffusion constant which is close to zero.

Figure 6B shows the data in Figure 6A for up to *d* = 10σ, with the additional systems of totally straight chains and chains without a bending term included in the plot. The curves indicate that *D*_∥_ < *D*_⊥_ from *d* = 2σ and up to about *d* = 10σ. As seen by Figure 4A, the chains fall into straighter conformations when the spacing is small. This is, as can be seen from Table 1, due to increased forces between the beads composing the chains. The pore space between the chains then take on a shape similar to channels oriented in the z-direction, opening for more rapid motion in the perpendicular direction. However, the proximity of the brush will still lead to collisions, lowering *D*_⊥_ compared to bulk. For motion parallel to the substrate, there are no such open channels, leading to a larger reduction of *D*_∥_ compared to *D*_⊥_. In summary, we expect *D*_∥_ < *D*_⊥_ for smaller values of *d* due to the anisotropic geometry of the system. Looking at Figure 4B, the chains appear to generate a more disordered structure for *d* = 10σ, and do not display similarly visible channels as they do for *d* = 3σ in Figure 4A. Inspecting Figure 6B further, we see that the difference between *D*_⊥_ and *D*_∥_ is larger for small *d*, where the channel-like formations are more prominent.

#### 4.3.2. Static brush

Figure 6 also shows *D*(*d*)/*D*_bulk_ for the static brush. As for the dynamic brush, *D*_∥_ < *D*_⊥_ for small values of *d*, and both *D*_⊥_ and *D*_∥_ converge to unity as *d* → ∞. Figure 6B shows that *D*_⊥, stat._ < *D*_⊥, dyn._ for smaller *d*, although *D*_⊥_ of the two system types agree within the standard deviation. This miniscule increase in the diffusion constant may be due to the flexibility of the dynamics chains, allowing the chains to sway due to the motion of the diffusing particle. This small difference in the diffusion constants of the static and dynamic brushes indicate that the dynamic flexibility of the chains do not play a major role in governing diffusion of free particles within the brush. The systems have similar geometry, as the chains have been made immobile only after equilibration. We therefore conclude that the geometry and not the dynamics of the brushes determines the diffusion coefficients.

#### 4.3.3. Other system types

Also included in Figure 6B are the diffusion constants of a free particle in a system where the bending potential energy (as defined by Equation (3)) is removed, and a system where the chains are straight and immobile. Due to the chain straightness in the latter system, we expect the channels to be more pronounced and that motion in the perpendicular direction will be less restricted. We therefore expect *D*_⊥, straight_ to be larger than *D*_⊥_ for any of the other systems, as demonstrated by Figure 6B. For simulations where the bending stiffness term is removed, the chains curl up more, yielding a slightly lower *D*_⊥_, as seen from the coral curve (marked by X's) in Figure 6B.

In the *z*-direction the geometry varies from clearly defined channels for the straight chains to mildly obstructed channels for the static and dynamic chains. In the direction parallel to the wall, along the *xy*-plane, there are few clear channels, but rather a forest of obstacles. Like the dynamic system, neither the static system nor the system without bending interactions exhibit ordered obstacles in the *xy*-plane. Although the straight system is more ordered than the other systems, it still exhibits the forest-like geometry in the *xy*-plane that all the system types display. We therefore expect that every system type possess similar values of *D*_∥_/*D*_bulk_. Figure 6B reveals that the *D*_∥_/*D*_bulk_ curves of the various system types all agree well within their standard deviations, unlike the curves of *D*_⊥_/*D*_bulk_.

### 4.4. Effective diffusion behavior

#### 4.4.1. Models for effective diffusion behavior

Many theoretical and experimental efforts strive to connect the diffusion constant to the porosity ϕ (Shen and Chen, 2007), that is, the fraction of space not occupied by solid material. In the simulated systems, the chain beads constitute the solid material, while the porosity is effectively the solvent volume fraction.

The porosity of our system can most easily be estimated through the total volume of the beads, *V*_beads_, and the volume of the system, *V*_system_. Using the volume of a sphere, $V_{\text{beads}}=N_{\text{chains}}N_{\text{bead}}\cdot \frac{4}{3}\pi a_{\text{f}}^{3}$, where *N*_chains_ is the number of chains, *N*_bead_ is the number of beads per chain and *a*_f_ is the radius of the beads. $V_{\text{system}}=N_{\text{chains}}d^{2}L_{z}$ was then found, where *L*_*z*_ is the average height of the chains. The porosity ϕ of the brush is hence


(12) $$\begin{array}{l} \phi =1-\frac{\frac{4}{3}\pi a_{\text{f}}^{3}N_{\text{bead}}}{d^{2}L_{z}}, \end{array}$$

However, since there are many porous systems with the same porosity, but with very different geometries, we expect the diffusion constant to depend on further properties of the porous system geometry. In our simulations, this is evident from comparing *D*_⊥_/*D*_bulk_ of the different system types, as seen in Supplementary Figure S6, where the system of straight chains displays a higher diffusion constant than the others for a wide range of ϕ's.

One property that affects the diffusion is the tortuosity τ, a measure of the degree to which diffusing particles are prevented to move in straight lines due to obstacles posed by the porous media. We may define τ as the path length Δ*l* traversed by the diffusing particle divided by the corresponding linear distance Δ*x* (Shen and Chen, 2007).


(13) $$\begin{array}{l} \tau =\frac{\Delta l}{\Delta x}. \end{array}$$

Using this definition, τ yields a correction to the bulk diffusion constant *D*_bulk_ that reads


(14) $$\begin{array}{l} D_{\text{eff}}=\frac{D_{\text{bulk}}}{\tau^{2}}, \end{array}$$

where *D*_eff_ is the effective diffusion constant in the porous medium. Shen and Chen (2007) list τ^2^ as a function of ϕ for several previous studies.

Having obtained the porosity ϕ, the diffusion constant *D*(*d*) can be compared to theory in order to characterize diffusion. Due to the anisotropic nature of the system, *D*_⊥_ and *D*_∥_ might be best described by different diffusion models. These models can subsequently be used to offer an approximation of *D*_⊥_ and *D*_∥_ for values of *d* that have not been simulated, but are within the range of interest. Furthermore, we can generalize from one bead size to another by using the parameters obtained in the fit in combination with Equation (12). Ultimately, this might prove useful in obtaining other net quantities from *D*, such as the capacitance.

The hyperbola of revolution listed in Shen and Chen is a system whose pore space comprises several consecutive hyperbolas (Petersen, 1958). Its tortuosity τ is defined through


(15) $$\begin{array}{l} \tau^{2}=2-\phi . \end{array}$$

Another expression for τ reads


(16) $$\begin{array}{l} \tau^{2}=\left(3-\phi \right)/2. \end{array}$$

This relation holds for various systems, such as for a dilute system of spheres (Akanni et al., 1987), spheres of various sizes that are allowed to overlap (Akanni et al., 1987) and randomly packed spheres of different radii (Neale and Nader, 1973). These systems are dubbed “ordered packings” by Shen and Chen (2007).

A tortuosity model frequently applied to heterogeneous catalysts was developed by Beeckman (1990), Yang et al. (2014), and Ghanbarian et al. (2013), where


(17) $$\begin{array}{l} \tau^{2}=\phi /\left[1-{\left(1-\phi \right)}^{1/3}\right]. \end{array}$$

The cation-exchange resin membrane consists of cross-linked polymers with uniformly distributed negative charges (Mackie and Meares, 1955). Its tortuosity was derived by Mackie and Meares (1955) using a random walk lattice model. A site in the model can either be occupied by polymer, solution or the walker. The walker is forced to move around polymer sites (Mackie and Meares, 1955), which it will encounter with a probability of *p*_first_ = ϕ_*p*_ = 1 − ϕ, where ϕ_*p*_ is the polymer fraction. Upon encountering a polymer site, there is a set probability *p*_next_ that the next site in the walk will also contain a polymer. This probability is chosen to reflect the properties of space in the cross-linked polymer and is found to be $p_{\text{next}}=\frac{1}{2}\left(2-\phi \right)$ by Mackie and Meares (1955). The probability that the walker will have to move two extra steps to get past the polymer is then $p_{1}=\left(1-\phi \right)\left[\frac{1}{2}\left(2-\phi \right)\right]$, the probability of three extra steps is $p_{2}=\left(1-\phi \right){\left[\frac{1}{2}\left(2-\phi \right)\right]}^{2}$ and so on. The total length Δ*l* the walker will have to move is related to the path length in bulk Δ*x* through Mackie and Meares (1955):


(18) $$\begin{array}{l} \tau =\frac{\Delta l}{\Delta x}=1+\left(1-\phi \right)\overset{\infty }{\underset{n=0}{\sum }}{\left[\frac{1}{2}\left(2-\phi \right)\right]}^{n}, \end{array}$$

where *n* is an index running over all the possible times the walker can hit polymer elements consecutively. Per definition, the pore fraction ϕ cannot exceed one, and for ϕ = 0 the system would only consist of polymer. Hence 0 < ϕ < 1, which means that $\frac{1}{2}\left(2-\phi \right)<1$ and that the geometric sum in Equation (18) converges. Performing the sum and adding the first term in Equation (18) yield (Mackie and Meares, 1955):


(19) $$\begin{array}{l} \tau =\frac{2-\phi }{\phi }. \end{array}$$

Combining Equations (14) and (12) with Equations (15)–(17) and (19) allows for a comparison between data and theories.

#### 4.4.2. Modified model for effective diffusion behavior

The probability *p*_next_ in Mackie and Meares was tailored to the cation-exchange resin membrane. However, the probability *p*_next_ can be modified to better capture the geometry of other systems such as the PNN system we study.

Keeping the probabilities *p*_first_ and *p*_next_ general for the sake of brevity, the probability that the walker will have to move two extra steps to get past the polymer is *p*_1_ = *p*_first_*p*_next_, the probability of three extra steps is $p_{2}=p_{\text{first}}p_{\text{next}}^{2}$ and so on. We thereby arrive at a more generalized option for Equation (18).


(20) $$\begin{array}{l} \tau =1+p_{\text{first}}\overset{\infty }{\underset{n=0}{\sum }}p_{\text{next}}^{n}, \end{array}$$

where *n* is an index running over all the possible times the walker can hit the polymer consecutively. As *p*_next_ is a probability and *p*_next_ = 0 corresponds to bulk behavior, 0 < *p*_next_ < 1. Performing a geometric sum on the last term in Equation (20) yields:


(21) $$\begin{array}{l} \tau =1+\frac{p_{\text{first}}}{1-p_{\text{next}}}, \end{array}$$

which offers a generalization to Equation (19).

Several different probabilities *p*_next_ were tested against the diffusion coefficients the polymer brush, and the best *p*_next_ was selected as a custom model. It was derived by assuming that if the walker hits a polymer section, it has a probability of *p*_next_ = *k* + (*k* − 1)(1 − ϕ) of not being able to make the next move because a neighboring site is occupied. Here, *k* is a tunable parameter. Since the solid is solely made up of polymer, the polymer fraction 1 − ϕ should equal the probability to first hit an obstacle, *p*_first_. The relation *p*_first_ = 1 − ϕ from Mackie and Meares (1955) should therefore hold for all systems and is kept as is. Insertion of *p*_first_ and *p*_next_ into Equation (21) yields an expression of the tortuosity:


(22) $$\begin{array}{l} \tau^{2}={\left[1+\frac{1-\phi }{1-k-\left(k-1\right)\left(1-\phi \right)}\right]}^{2}. \end{array}$$

This model will be referred to as the custom model. Combining Equations (14) and (12) with Equation (22) allows for a comparison between data and model.

#### 4.4.3. Power law model

Judging by the shape of the curves in Figure 6, we may approximate *D*/*D*_bulk_ as a power law:


(23) $$\begin{array}{l} \frac{D}{D_{\text{bulk}}}=1-d^{-n}c, \end{array}$$

where *n* and *c* are tunable parameters for this model.

#### 4.4.4. Comparison with data

**Figure 7:**
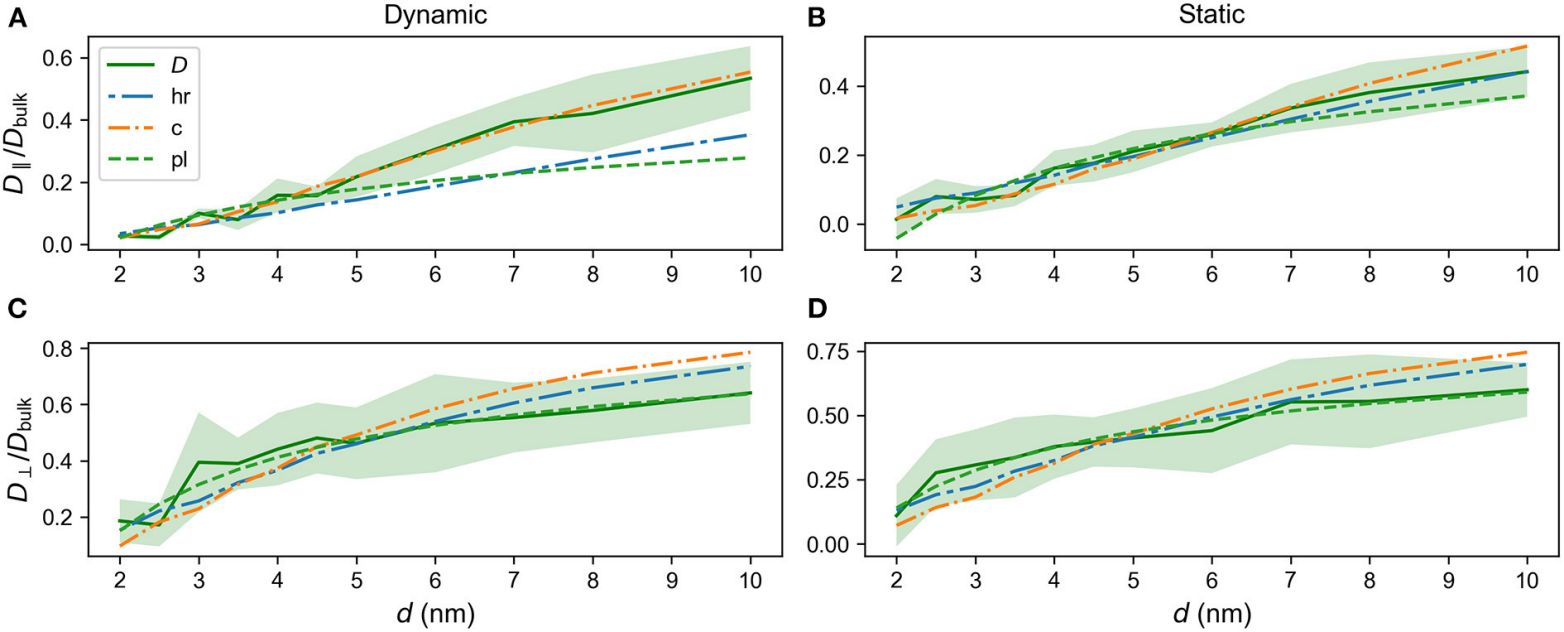
Diffusion constants as a function of *d* together with fits. Solid line-*D*_∥_/*D*_bulk_ and *D*_⊥_/*D*_bulk_ vs. *d* for dynamic and static brushes. **(A)** *D*_∥_/*D*_bulk_ for the dynamic brush. **(B)** *D*_∥_/*D*_bulk_ for the static brush. **(C)** *D*_⊥_/*D*_bulk_ for the dynamic brush. **(D)** *D*_⊥_/*D*_bulk_ for the static brush; *D*, data; hr, Hyperbola of revolution fit; c, Custom model fit; pl, Power law fit. Standard deviations are indicated by shaded regions. Parameters of the fits are listed in Table 2.

As none of the models listed in Shen and Chen (2007) closely resemble the brush systems we study, the bead size *a*_f_ was left as a free parameter for the curve fit using the Levenberg-Marquardt algorithm through SciPy's optimize.curve_fit function (Virtanen et al., 2020), as the theoretical models were not tailored to the brush and some discrepancy should be expected. The optimal parameter values for each fit to the diffusion constant of a neutral particle in the dynamic brush and the static brush from Figure 7 is given in Table 2, together with the relative differences between the parameters in the parallel and perpendicular directions. The parameter *k* in the custom model was found by the Trust Region Reflective algorithm as implemented in SciPy and *n* and *c* for the power law was found by the Levenberg-Marquardt algorithm as implemented in SciPy. The results are listed in Table 3 for all directions for the static and dynamic brush. The brush regime is of interest for the fits since this configuration should correspond to developed PNNs (Richter et al., 2018). Furthermore, the PNNs are reported to be quite dense (Deepa et al., 2006). The range *d* = 2 − 10σ was chosen to satisfy as closely as possible both these criteria. Figure 7 shows *D*_∥_/*D*_bulk_ and *D*_⊥_/*D*_bulk_ for the dynamic and static brushes together with fits. For readability purposes, only three models are selected for plotting: The hyperbola of revolution, which returns the same curves as the ordered packings, the custom model and the power law model. The complete set of fits including different custom models are shown in Supplementary Figures S7–S10 and specified in Supplementary Tables S2, S3. The cation-exchange resin model proved a poor fit in all instances, while the heterogeneous catalyst was slightly outperformed by the power law for *D*_⊥_, while providing a poor fit for *D*_∥_.

**Table 2:** Parameters *a*_f_ of the fits to models listed in Shen and Chen (2007) for the dynamic and static brush.

	**op**	**hr**	**hc**	**rm**
**Dynamic brush**				
*a*_f,∥_ (nm)	3.97 ± 0.11	3.15 ± 0.09	4.77 ± 0.23	15.89 ± 0.10
*a*_f,⊥_ (nm)	2.32 ± 0.13	1.83 ± 0.10	1.32 ± 0.13	21.4 ± 0.6
$\frac{a_{\text{f},∥}-a_{\text{f},⊥}}{a_{\text{f},∥}}$	0.42	0.72	0.42	−0.34
**Static brush**				
*a*_f,∥_ (nm)	3.55 ± 0.12	2.82 ± 0.10	2.81 ± 0.18	17.55 ± 0.25
*a*_f,⊥_ (nm)	2.47 ± 0.15	1.96 ± 0.12	1.39 ± 0.16	21.3 ± 0.7
$\frac{a_{\text{f},∥}-a_{\text{f},⊥}}{a_{\text{f},∥}}$	0.30	0.30	0.51	-0.21

op, ordered packings; hr, hyperbola of revolution; hc, heterogeneous catalyst; rm, cation-exchange resin membrane.

**Table 3:** Parameter *k* of the fit to the custom model and parameters *n* and *c* of the fit to the power law.

	**Dyn. ∥**	**Dyn. ⊥**	**Stat. ∥**	**Stat. ⊥**
*k*	0.9811 ± 0.0011	0.947 ± 0.007	0.9829 ± 0.0014	0.957 ± 0.007
*n*	0.189 ± 0.22	0.53 ± 0.12	0.31 ± 0.05	0.46 ± 0.14
*c*	1.116 ± 0.019	1.22 ± 0.12	1.29 ± 0.08	1.18 ± 0.14

Note that within more than 2–3 standard deviations, *k* does not reach one, guaranteeing the numerical stability of Equation (22). Dyn., dynamic brush; stat., static brush.

Figure 7A reveals that the power law of Equation (23) offers a poor fit to *D*_∥_/*D*_bulk_ for the dynamic brush. A better fit to the data is provided by the hyperbola of revolution. The custom model overall offers the best fit to the data, although it starts to deviate slightly for larger values of *d*. This might be an artifact of the data set, since there are relatively fewer data points for large *d* compared to small *d*.

In Figure 7B *D*_∥_(*d*)/*D*_bulk_ for the static brush is plotted together with fits to the different models. The best parameter fits for the static brush are given in Table 2 along with the relative differences between the results in the parallel and perpendicular directions. The parameters *a*_f_ are consistently larger for parallel motion, with the exception of the cation-exchange resin membrane model. The custom model and the power law provide fits of comparable quality, with both models deviating from the data set for larger *d*'s and the custom model providing a better fit at smaller *d*'s. Both fits mostly stay within the standard deviation. The hyperbola of revolution more closely aligns with the data set. Considering the unrealistically large value of *a*_f_ for the hyperbola of revolution in Table 2, the custom model proves the best model overall for *D*_∥_(*d*)/*D*_bulk_ in the static brush.

As seen by comparing Figures 7A,C, *D*_⊥_/*D*_bulk_ for the dynamic brush generally has larger uncertainties than *D*_∥_/*D*_bulk_ for *d* ∈ [2σ, 10σ]. The fitted parameters are therefore more uncertain. Both the custom model and the hyperbola of revolution perform poorly on this system, overestimating the diffusion constant for larger *d* and slightly underestimating it for smaller *d*. The power law more closely aligns with the data points, staying within a standard deviation while retaining a similar curvature. Similar results are found for *D*_⊥_/*D*_bulk_ for the static brush, as shown in Figure 7D.

It is worthwhile to note that the theoretical models were originally developed for describing diffusion in three-dimensional space. By comparing to two-dimensional diffusion in the case of *D*_∥_ and one-dimensional diffusion in the case of *D*_⊥_, the models lose some of their interpretive qualities. Instead, they provide a heuristic mathematical framework. The power law often takes on this role, as it is abundant in nature (Gheorghiu and Coppens, 2004).

### 4.5. Relation between *D*_⊥_ and *D*_∥_

**Figure 8:**
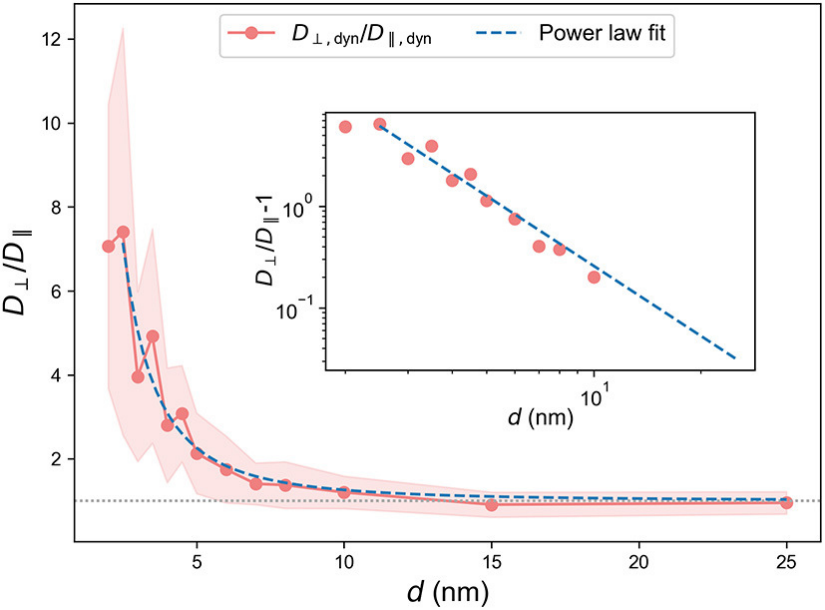
*D*_⊥_/*D*_∥_ vs. *d* for the dynamic brush, together with a fit to the power law *Ad*^−*l*^ + 1. Insert: A log-log plot of *D*_⊥_/*D*_∥_ − 1 together with *Ae*^−*d*/*l*^. The fit is performed in the range *d* ∈ [2.5σ, 25σ]. Standard deviations are indicated by shaded regions. Insert: A log-log plot of *D*_⊥_/*D*_∥_ − 1 and *Ad*^−*l*^. Note that *D*_⊥_/*D*_∥_ − 1 fell below zero for *d* = 15σ and *d* = 25σ so that these points are not visible in the log-log plot.

Both diffusion constants, *D*_⊥_ and *D*_∥_, approach *D*_bulk_ when the spacing *d* becomes large. However, the way they approach *D*_bulk_ can provide insights into the role of anisotropy in the system. To address this, we plot the ratio *D*_⊥_/*D*_∥_ as a function of spacing *d* in Figure 8. For an isotropic system, we would expect the diffusion constants to depend on *d*, but the ratio to be constant. Figure 8 shows that *D*_⊥_/*D*_∥_ approaches unity for large *d*. The behavior of *D*_⊥_/*D*_∥_ is well approximated by a power law, $D_{⊥}/D_{∥}=Ad^{-l}+1$, as illustrated by the plot of the fitted function in Figure 8 and in the log-log plot insert. We performed similar analysis for all systems (see Supplementary Figures S11–S13) and the results are shown in Table 4. The range of spacings, *d*, over which the systems display significant effects of anisotropy is within the range relevant for PNNs (Deepa et al., 2006; Richter et al., 2018). We therefore expect anisotropic effects in diffusion to be important for PNNs.

**Table 4:** Parameters of the fits of *D*_⊥_/*D*_∥_ to *f*(*d*) = *Ad*^−*l*^ + 1 for the different system types.

**Brush type**	**Dynamic**	**Static**	**Straight**	**No stiffness**
*A*	49.9 ± 15.6	26.8 ± 6.7	104.1 ± 22.1	18.9 ± 1.7
*l* (σ)	2.29 ± 0.28	2.03 ± 0.27	2.15 ± 0.14	2.10 ± 0.11
*d*_start_ (σ)	2.5	2	4	2

The last line indicates the start *d*_start_ of the fit, which is performed on the interval *d* ∈ [*d*_start_, 25σ].

### 4.6. Diffusion vs. particle size

Simulations were performed for a diffusing particle of various radii *a* in the dynamic brush. The resulting perpendicular diffusion constants were divided by *D*_⊥_ for *a* = 0.5 nm to illustrate how diffusion is affected by particle size.

Scaling theories of diffusion in polymer liquids predict that the diffusion constant scales with particle diameter σ_LJ_ as *D* ∝ 1/σ_LJ_ when σ_LJ_ < ξ (Cai et al., 2011), where ξ is the correlation length. ξ is defined the average distance from one chain bead to the closest bead on another chain (Cai et al., 2011). We found ξ by looping over all beads for the last time frame of the simulation and finding the closest bead on another chain. The average was taken over all beads and realizations. A plot of ξ vs. *d* is presented in Supplementary Figure S14, revealing that σ_LJ_ < ξ for the majority of spacings studied. *D*_⊥_/*D*_⊥, *a* = 0 nm_ is therefore compared to σ_LJ,*a* = 0 nm_/σ_LJ_ = 1/2*a*.

**Figure 9:**
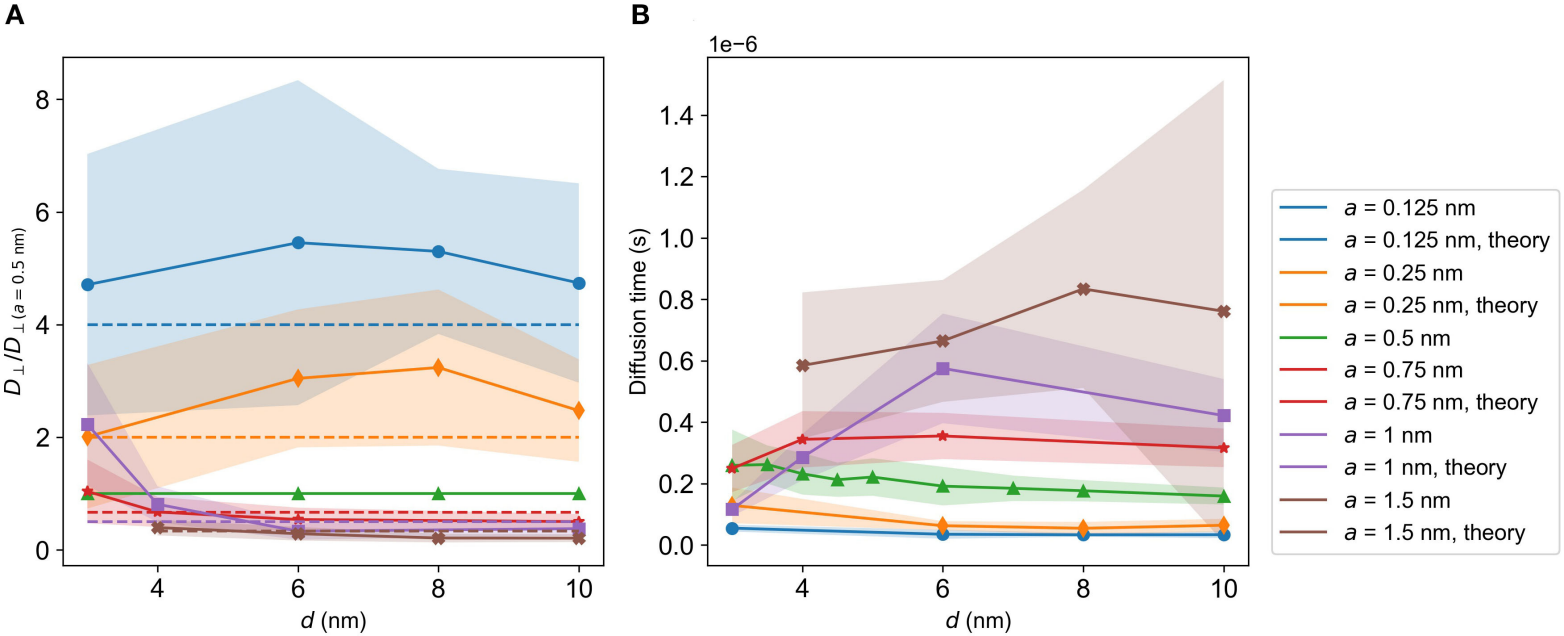
Diffusion properties for an uncharged particle of different radii *a* in a dynamic brush. **(A)** *D*_⊥_/*D*_⊥(*a* = 0.5nm)_ vs. *d*, where *D*_⊥(*a* = 0.5 nm)_ is *D*_⊥_ for *a*= 0.5 nm. The dashed lines indicate the expected scaling of the diffusion constant as presented in Cai et al. (2011). **(B)** Diffusion times through the brush vs. *d* for a brush height of *h* = 500 nm. The curves were found through application of Equation (10).

Results showed an acceptable agreement with theory, as shown in Figure 9A. *D*_⊥_ increases with decreasing *a*, as expected from Equation (6). The exception is for particle diameters close to *d*, i.e., when the brush is dense compared to particle size. Inspection of trajectories reveals occasional extrusion of the particle from the brush for such parameter combinations, probably due to unfavorable initial conditions for these large particles.

We applied (Equation 10) to find the corresponding diffusion time through a brush of height *h* = 500 nm as a function of spacing *d*, as shown in Figure 9B. The diffusion time stays within 0.4 μs for *a* ≤ 0.5 nm.

### 4.7. Diffusion of charged particles

#### 4.7.1. Diffusion constants

**Figure 10:**
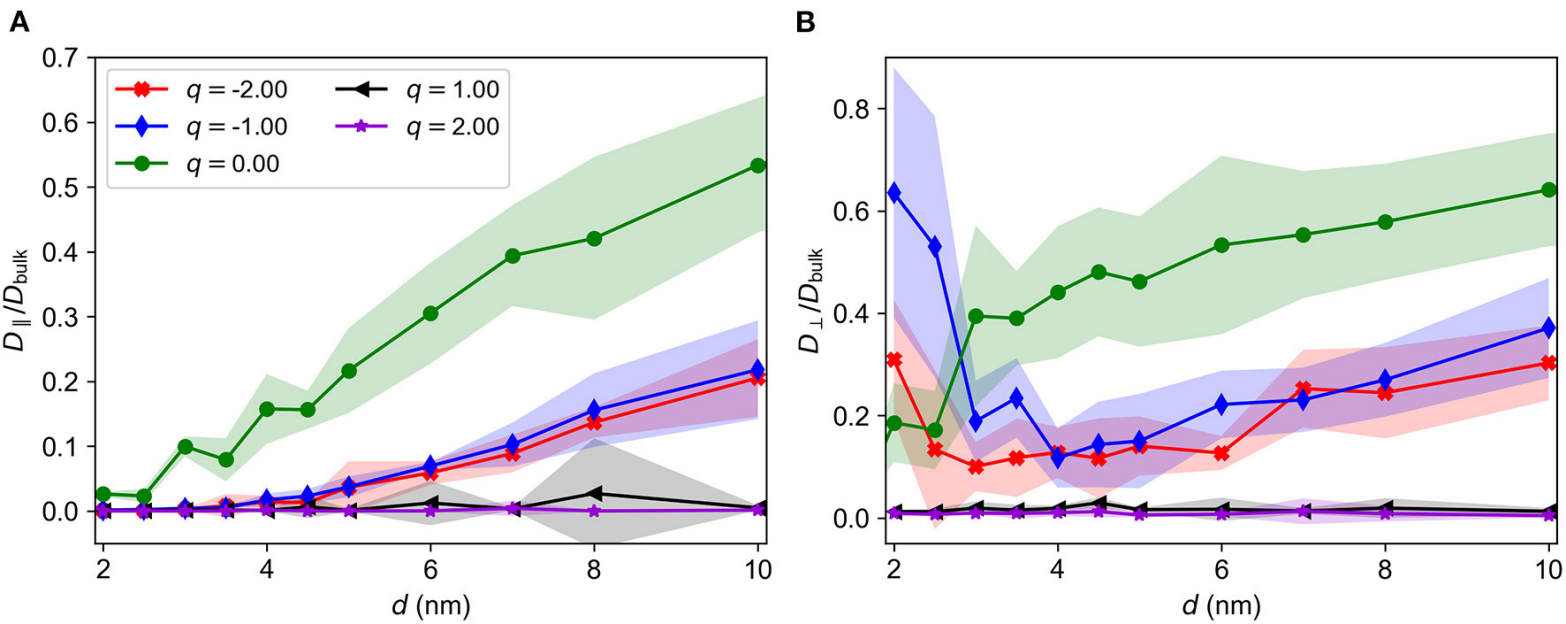
*D*/*D*_bulk_ vs. *d* for a diffusing particle of charge *q*. **(A)** *D*_∥_/*D*_bulk_ vs. *d*. **(B)** *D*_⊥_/*D*_bulk_ vs. *d*. Standard deviations are indicated by shaded regions.

Ionic transport and the diffusion of molecules with more complex surface charge distributions are important in PNNs. We therefore address the diffusion of charged particles, which may represent an ion or a charged molecule, through the brush as a model for transport through PNNs. Figure 10A shows *D*_∥_/*D*_bulk_ vs. *d* for diffusing particles with different charges. We see that the diffusion constant is very small for positively charged particles compared to negatively charged and neutral particles due to electrostatic interactions. Positively charged particles are therefore subject to an intermittent and slow transport mechanism, jumping between trapping sites that are close to the chain beads (see Supplementary Videos S2–S4) in a process similar to cations binding to negatively charged sites on GAG chains in the PNNs (Morawski et al., 2015). Negatively charged diffusing particles experience repulsive electrostatic forces from the chain beads, which effectively increases the particle interaction size, and therefore restricts the available pore space and lowers of the diffusion constant.

Figure 10B show that the behavior of *D*_⊥_/*D*_bulk_ for charged particles is similar to that of *D*_∥_/*D*_bulk_. The increased value of *D*_⊥_ for small *d* (*d* < 3.5σ) is likely an artifact from the simulation procedure: If the initial position of the charged particle had been outside of the brush, the repulsive electrostatic forces would have most likely have prevented it from entering the brush. Lower concentrations of anions compared to cations have been observed in GAG-rich environments (Morawski et al., 2015), so it seems reasonable that the brush should exclude anions at sufficiently small *d*. Overall, the diffusion constants are reduced by a factor of two when the charge was increased from *q* = 0 to *q* = −*e*.

#### 4.7.2. Conductivity, resistivity, and resistance

The conductivity can be computed from the diffusion constant of ions in solution through


(24) $$\begin{array}{l} \sigma_{e}=\frac{F^{2}}{RT}\underset{x}{\sum }D_{x}z_{x}^{2}\left[x\right], \end{array}$$

where σ_*e*_ is the electrical conductivity, *F* is Faraday's constant, *R* is the gas constant, *D*_*x*_ is the diffusion constant of ionic species *x*, *z*_*x*_ is the valency of the ion and [*x*] is its concentration (Qian and Sejnowski, 1988). To find the conductivity σ_*e*_, we used concentrations from Raimondo et al. (2015), adding together concentrations of ions of the same valency for use in Equation (24).

**Figure 11:**
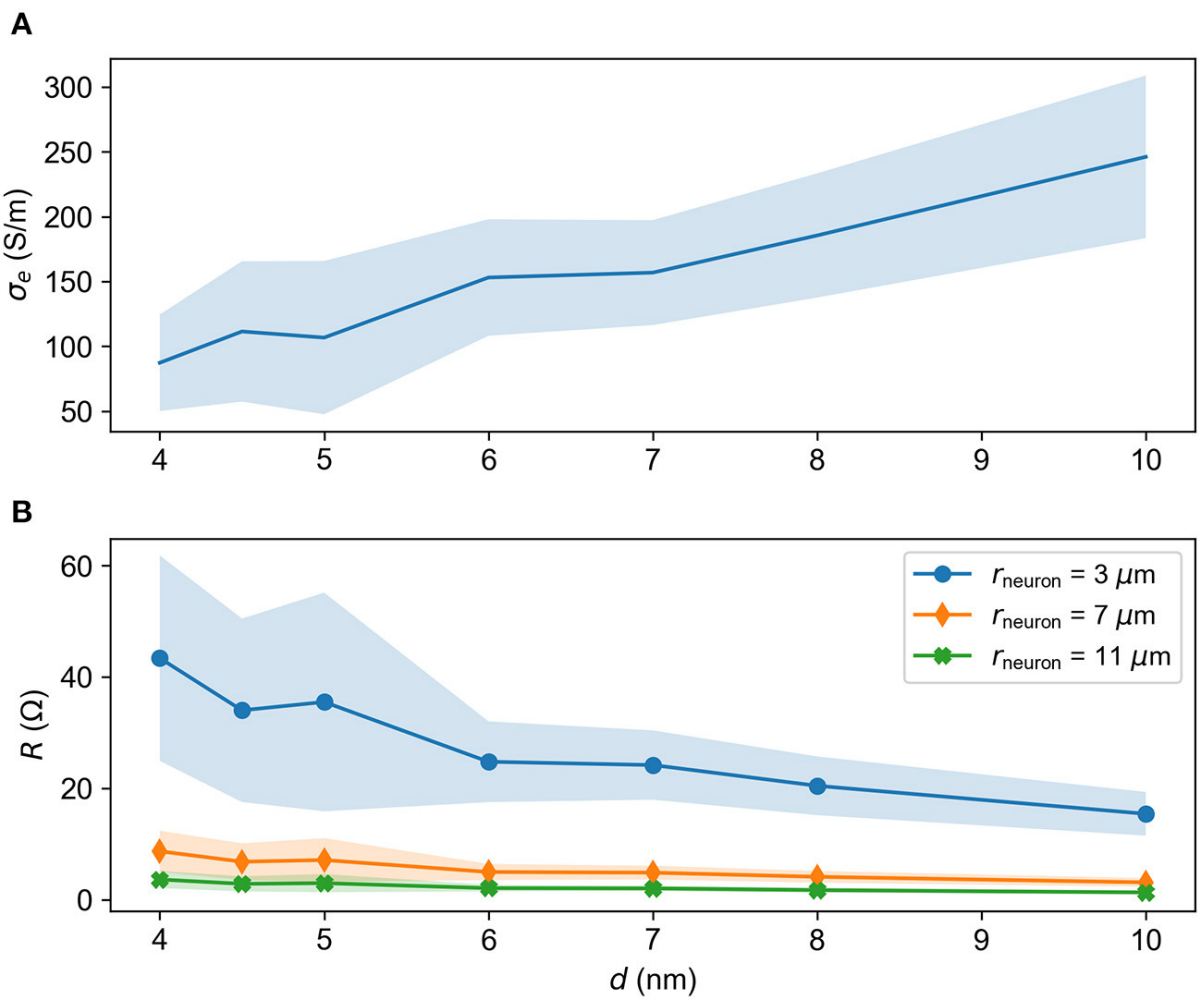
Electrical properties of the brush as a function of *d*. **(A)** Electrical conductivity σ_*e*_(*d*) through the brush. **(B)** Resistance *R* vs. *d* for different cell radii *r*_neuron_ assuming a brush height of *h* = 500 nm. Standard deviations are indicated by shaded regions.

Figure 11A shows the electrical conductivity σ_*e*_ as a function of *d* across the brush. Here, we have excluded small values of *d* since these systems are unrealistic for charged particles as argued above.

The resistance *R* is connected to the resistivity ρ_*e*_ = 1/σ_*e*_ by


(25) $$\begin{array}{l} R=\frac{\rho_{e}l}{A} \end{array}$$

where *l* is the length of the conducting material and *A* is its cross-section. In cases where *A* varies along the length of the conductor, the equation should be integrated in order to yield correct results. PNNs wrap around the soma and proximal dendrites of a neuron (van 't Spijker and Kwok, 2017). Assuming that PNNs take the form of a spherical shell of thickness *h*, the resistance of the nets become:


(26) $$\begin{array}{l} R=\frac{\rho_{e}}{4\pi }\left(\frac{h}{r\left(r+h\right)}\right) \end{array}$$

where *r* is the radius of the neuron. The resistance is inversely proportional to the diffusion constant, as seen from Equation (24), meaning that a lowered diffusion constant leads to an increased resistance.

Figure 11B shows the resistance *R* vs. *d* for a brush with height *h* = 500 nm for different cell radii *r*_neuron_ (Quan et al., 2013). We see that *R* decreases with increasing *d*, which is expected because as the brush becomes sparser, the average interactions between brush and particles decrease.

We found the effective resistance of the brush to be *R* ≃ 1 − 62 Ω. Input resistances of neurons are usually much higher, on the order of magnitude of 100 MΩ (Jouhanneau et al., 2018). With their small resistance, the brushes do not appear to pose a significant barrier to current of negatively charged ions.

## 5. Discussion

We have used *D*_∥_/*D*_bulk_ and *D*_⊥_/*D*_bulk_ to characterize diffusion inside a brush as model for transport in PNNs. The values of *D*_⊥_/*D*_bulk_ in Figure 6 show that a charged brush limits diffusion in the transverse direction as mentioned by van 't Spijker and Kwok (2017), an effect that increases with increased density. Low values of *D*_⊥_ in dense brushes supports that glycosaminoglycans block out molecules that may be potentially harmful to the cell (Suttkus et al., 2014; Morawski et al., 2015). Lateral diffusion, as illustrated by *D*_∥_/*D*_bulk_ in Figure 6, exhibit a similar behavior.

Negatively charged free particles have a lower diffusion constant than uncharged particles, highlightning the ability of the net to act as a barrier to ions, as has been proposed by others Morawski et al. (2015); van 't Spijker and Kwok (2017). The drastically lowered diffusion constants of positively charged particles as shown in Figure 10 are to be expected as the positive charges experience an attraction to the negatively charged nets by electrostatic forces. However, this may only be a transient effect until the negative charge has been neutralized by trapped positively charged ions.

Equation (22) represented a good heuristic model for diffusion in this system. It is based on the generalized version of the Mackie and Meares (1955) model that we introduced in Equation (21), which could prove a useful model for the diffusion constant in a wide range of systems. The parameter *p*_next_ can be estimated for a specific geometry, providing a method to predict τ for given geometries.

The viscosity η in Equation (6) was assumed to be constant and equal that of bulk. However, when the diameter of the diffusing particle is larger than the correlation length ξ, an effective viscosity arises in polymer liquids (Cai et al., 2011). This viscosity is increased compared to bulk (Cai et al., 2011). Effective viscosities have also arisen in polymer brushes (Doyle, 1998). In the present study, the particle diameter σ_LJ_ < ξ for all *d*, and the same holds for all data points presented in Figure 9, as seen by Supplementary Figure S14.

Representing the perineuronal net structure by a charged planar brush is a strong simplification. As seen by Figure 1, perineuronal nets consist of several components that vary in shape and size, which are bound and cross-linked to form a complicated structure for which the details are currently not known. An important aspect of PNNs is the highly negative charge density of the GAGs (Richter et al., 2018). Since we have included a high charge density in our model, the diffusional behavior should still capture key aspects of motion into and out of the perineuronal nets.

Relatively small values of *d* were used in this study compared to what should be realistic for PNNs. Real-life PNNs will have side CSPGs attached, akin to side chains. The CSPGs have a certain spatial extent, increasing the effective volume of each HA chain in the nets. A model consisting of linear chains correspondingly underestimates the volume fraction of polymer inside the brush. Lowering *d* will therefore yield a more accurate porosity, which is an important concept in diffusion in porous media. Given that accurate measurements of grafting densities and CSPG spacing on HA in PNNs are not currently available, this approach seemed favorable over creating a detailed, more realistic model with uncertain parameters. We simulated systems with a wide range of spacings, but emphasis was placed on the range *d* = 2 − 10 nm for which diffusion was significantly different than in bulk. This range was chosen as PNNs are hypothesized to restrict transport (Morawski et al., 2015; van 't Spijker and Kwok, 2017; Fawcett et al., 2019).

Estimates of persistence lengths of each GAG vary somewhat, with persistence lengths as short as 4 nm being measured for HA (Bano et al., 2016). The persistence length is governed by the relative size of the bending interaction parameter *K*_bend_ in Equation (2). This force field term was absent in the system type with no stiffness, meaning that it should have a persistence length close to the length of the bead, that is 1 nm. The stiffness of the chains did not affect diffusion noticeably, as seen by comparing the diffusion constant in systems with bending terms (dynamic) and without (no stiffness) in Figure 6B. Hence, the exact value of *l*_*p*_ does not pertain significantly to the study of diffusion constants in these brushes.

PV interneurons, around which PNNs frequently enwrap, are well-known to have low input resistance (Ferguson and Gao, 2018; Jouhanneau et al., 2018). If the net resistance was large or comparable to the membrane resistance, the total resistance of PV interneurons would be large compared to other neurons, or in the very least not significantly lower. The low resistance of our simplified PNN model is hence in agreement with experimental results, but may have corrections due to the simplifications made.

The PNNs are reported to protect neurons against ions and molecules of different sizes (van 't Spijker and Kwok, 2017), so particles smaller than the chain beads will sometimes be of interest. The Lennard-Jones potential (Equation 1) is included to account for steric interactions, and σ_LJ_ and hence *r*_*c*, LJ_ should therefore depend on particle size. A smaller particle will thus be less influenced by the Lennard-Jones potential as it is within the cutoff for a smaller amount of time. Furthermore, the mass should be adjusted according to size, yielding a smaller particle mass. Taking both radius and mass into account, the damping term and the random term in Equation (6) will be lower. The motion of the particle will therefore be faster, and the diffusion constant in brush and in bulk will be a bit higher. This is in agreement with Figures 3, 9.

The charged particle is sensitive to the Debye interaction, which does not depend on particle size, only separation distance. When decreasing the particle radius, the Lennard-Jones interaction will be weakened compared to the Debye interaction. Judging by Figure 10, the Debye interaction already has a large effect on the diffusion constants for *a*= 0.5 nm, so we expect further reductions in the ratio of the Lennard-Jones interaction to the Debye interaction to have a limited effect on particle diffusion. Smaller particles should therefore display moderately reduced diffusion constants. A moderate decrease in diffusion constant would lead to a moderately heightened resistance, as seen by Equations (24) and (26).

The size of the Debye interaction depends on the charges involved. CS in the brain tends to be singly sulfated and consequently has twice the charge of HA (Logsdon et al., 2022). One step in moving toward a more detailed model of PNNs could therefore include higher charges on the chain beads, as CSPGs are attached to HA. An increased bead charge will increase the strength of the Debye interaction, as seen by Equation (4). Similarly, the Debye strength was increased in Section 4.7, where the charge of the particle was varied. As seen from Figure 10, the diffusion constant did not depend strongly on particle charge and hence the size of the Debye interaction, though charged particles exhibited stronger diffusion constants than neutral ones. These findings indicate that the exact strength of the Debye interaction is not a strong determinant of diffusion.

However, the polymer chains will experience a stronger interchain repulsion upon an increased bead charge, giving rise to a somewhat more stretched conformation of the chains. As seen in Figure 6, stretching of chains leads to an increase in the diffusion coefficient perpendicular to the wall. For the charged particle, this will decrease the resistance. Figure 6 and Supplementary Figure S6 indicate that the specific geometry of the system plays a relatively small role in diffusion. As the Debye interaction decays quickly with separation, it is fair to assume that the stretching of the chains should be rather moderate. We therefore only expect a slight increase in the diffusion constant and decrease in resistance when the bead charge is increased.

Another simplification made in the model is that only one charged particle diffuses within the brush. The ionic concentration in the brain is on the order of magnitude of 100 mM (Raimondo et al., 2015), meaning that ions present in the fluid interact with each other. This interaction can affect transport, most likely lowering the diffusion constant as the particle is subject to forces from a lot of other particles, effectively slowing it down (Sterratt et al., 2011). The resistance would then be increased. Given the order of magnitude difference between the brush resistance and the membrane resistance of a neuron, we believe that neither of the additional effects discussed should increase the PNN resistance to an extent that it is comparable to the membrane resistance.

## 6. Conclusion

Diffusion was characterized for a freely diffusing particle in a system of negatively charged polymers tethered to a substrate, a simplified model of the perineuronal nets. The anisotropic nature of the system yielded different diffusion constants in directions parallel and perpendicular to the substrate for denser brushes. Both diffusion constants displayed a clear reduction compared to bulk, particularly for smaller brush spacings. This finding supports the notion that PNNs restrict transport.

Isotropic diffusion was recovered in the limit of very dilute brushes, where the system is close to bulk. In order to characterize how the system approached bulk, the ratio of the diffusion constants were fit to a decaying power law.

To study the effect of brush geometry and dynamics on diffusion, several different system types were probed. Altering the dynamics of the brush yielded only small differences in diffusion of the free particle, with the diffusion constants of static brushes and dynamic brushes with and without a bending term all agreeing within the standard deviation. The free particle diffusion constant in a system of straight, fixed chains yielded a higher diffusion constant in the perpendicular direction than for the other systems, while the parallel diffusion constant remained the same within the standard deviation. In summary, the exact dynamics of the brush did not affect the diffusion constants noticeably.

The behavior of each diffusion constant with increasing spacing *d* was compared to existing theories for intermediate values of *d* in a heuristic manner. The hyperbola of revolution and ordered packings models provided the best fit of the theoretical models considered to the diffusion constant as a function of porosity. A modified version of the cation-exchange resin model was implemented and displayed an acceptable performance. A power law proved the best fit to *D* vs. *d* in the perpendicular direction for both system types and was used to estimate the diffusion time through a *h* = 500 nm brush.

Simulations were performed for different particle charges and combined with theory to obtain electrical properties of the brush. The resistance of the brush was found to be orders of magnitude smaller than the resistance of a neuron membrane, implying that the PNNs should not affect the resistance of a cell to a large extent.

## Data availability statement

The datasets presented in this study can be found in online repositories. The names of the repository/repositories and accession number(s) can be found below: https://doi.org/10.5281/zenodo.7304745.

## Author contributions

KH performed simulations and analysis. AM-S and KH contributed equally to modeling, concepts and writing. All authors contributed to the article and approved the submitted version.

## Funding

This study was funded by The Research Council of Norway (https://www.forskningsradet.no/en/) grant no. 568117 to KH and AM-S.

## Conflict of interest

The authors declare that the research was conducted in the absence of any commercial or financial relationships that could be construed as a potential conflict of interest.

## Publisher's note

All claims expressed in this article are solely those of the authors and do not necessarily represent those of their affiliated organizations, or those of the publisher, the editors and the reviewers. Any product that may be evaluated in this article, or claim that may be made by its manufacturer, is not guaranteed or endorsed by the publisher.
