# Supplementary material for: Perineuronal nets restrict transport near the neuron surface: A coarse-grained molecular dynamics study
Source: Front Comput Neurosci. 2022 Nov 17;16:967735. doi: 10.3389/fncom.2022.967735 (PMC9714573; doi:10.3389/fncom.2022.967735)
Supplement: Supplementary file 1 [file Data_Sheet_1.PDF]

Supplementary Material

1 SUPPLEMENTARY VIDEO DESCRIPTION

S1 Video

The particle gets trapped within the brush for $d = 1.5\sigma$. Periodic images are omitted. The particle coordinates are not wrapped back into the original simulation box.

S2-S4 Video

The particle of charge $q = e$ experiences an attraction to the negatively charged chain beads in a brush. Periodic images are omitted. The particle coordinates are not wrapped back into the original simulation box. S2: $d = 3\sigma$, S3: $d = 4\sigma$, S4: $d = 6\sigma$.

2 SUPPLEMENTARY TABLES AND FIGURES

2.1 Figures

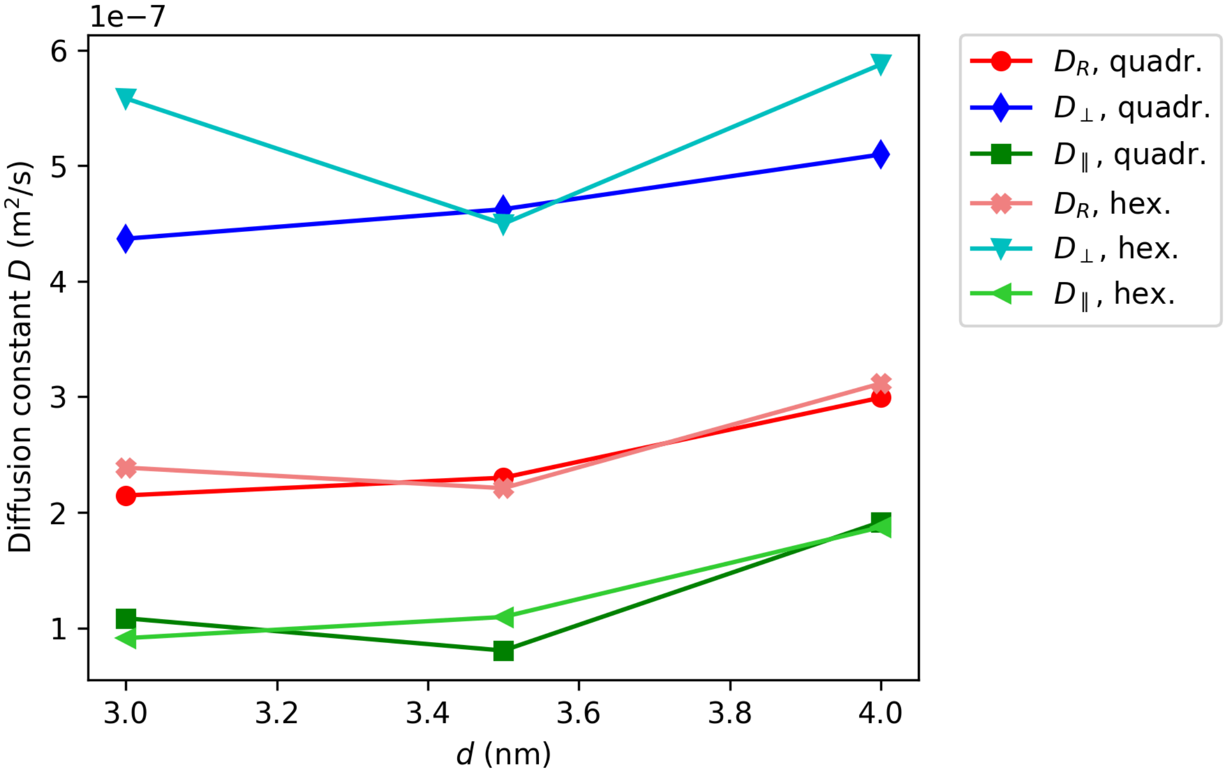

Figure S1. Diffusion constants for brushes of quadratic and hexagonal grids. The grid structure does not affect the diffusion constants. D_R - total diffusion constant; $D_\perp$ - diffusion constant in the z-direction; $D_\parallel$ - diffusion constant in the xy-plane; quadr. - Quadratic grid structure; Hex - Hexagonal grid structure.

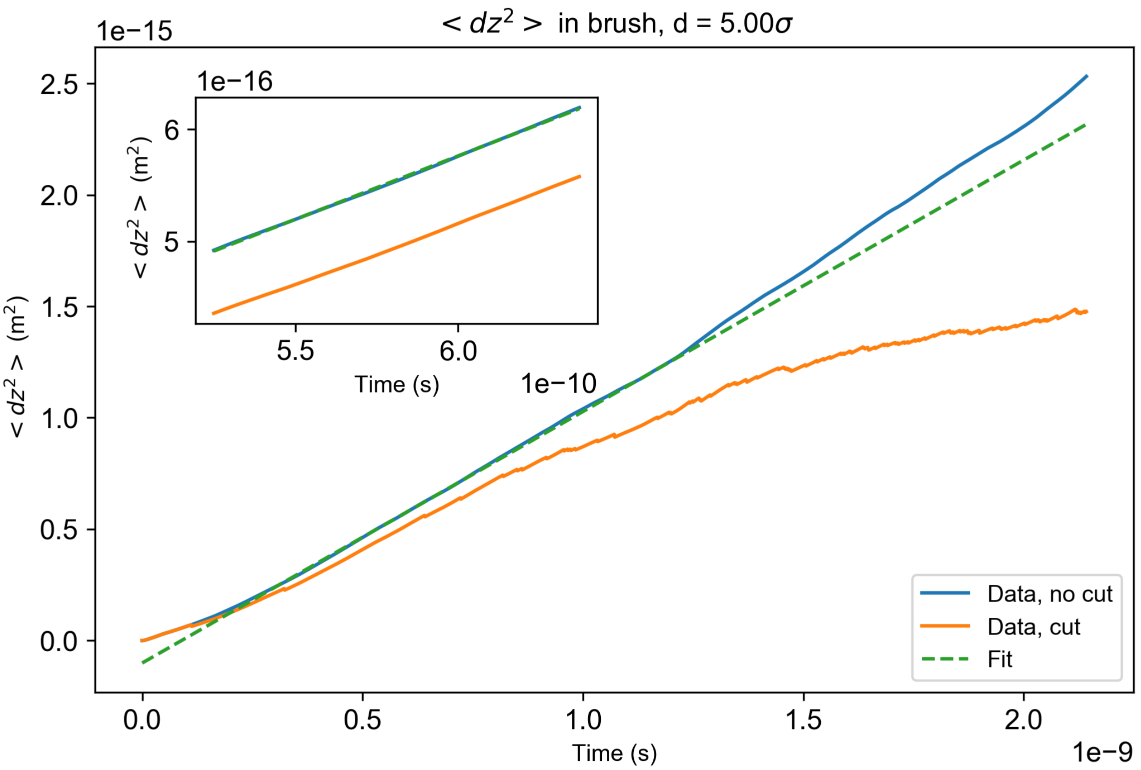

Figure S2. An illustration of how the diffusion constant $D_\perp$ is found from the mean squared displacement (MSD). This example shows $d = 5\sigma$. The figure shows the MSD for the whole of the interval, while the inset shows the MSD on the interval used to determine $D_\perp$. The dashed line is the fit to a straight line, whose slope is used to determine $D_\perp$. Data, no cut - MSD for all particles; Data, cut - MSD for the particles still in the brush at each point in time; Fit - the fit to the straight line. Note that in the inset, the fit overlaps almost completely with the MSD for all particles.

2.2 Tables

Table S1. Prefactors A in the relation $L_z = Ad^{-2/3}$ between brush height L_z and brush spacing d. A_{Fit} is the prefactor of the fit to the data points, while A_{BP}, A_{PP} and A_{ST} are the expected prefactors from different theories. Dyn. - dynamic brush; Stat. - static brush; No stiff. - brush without bending stiffness. Mean field approximations: BP - Box profile; PP - Parabolic profile; ST - scaling profile. Mathematical expressions and descriptions of the different mean field approximations can be found in e.g. (Attili et al., 2012). Note that A_{ST} is approximate as it depends on a prefactor of order unity that is a priori unknown. Note also that the expected height of the brush without bending stiffness differs from that of the other systems due to a shorter persistence length.

	Dyn.	Stat.	No stiff.
A_{Fit}	223	220	191
A_{BP}	188	188	139
A_{PP}	253	253	186
A_{ST}	271	271	200

REFERENCES

Attili, S., Borisov, O. V., and Richter, R. P. (2012). Films of End-Grafted Hyaluronan Are a Prototype of a Brush of a Strongly Charged, Semiflexible Polyelectrolyte with Intrinsic Excluded Volume , 12

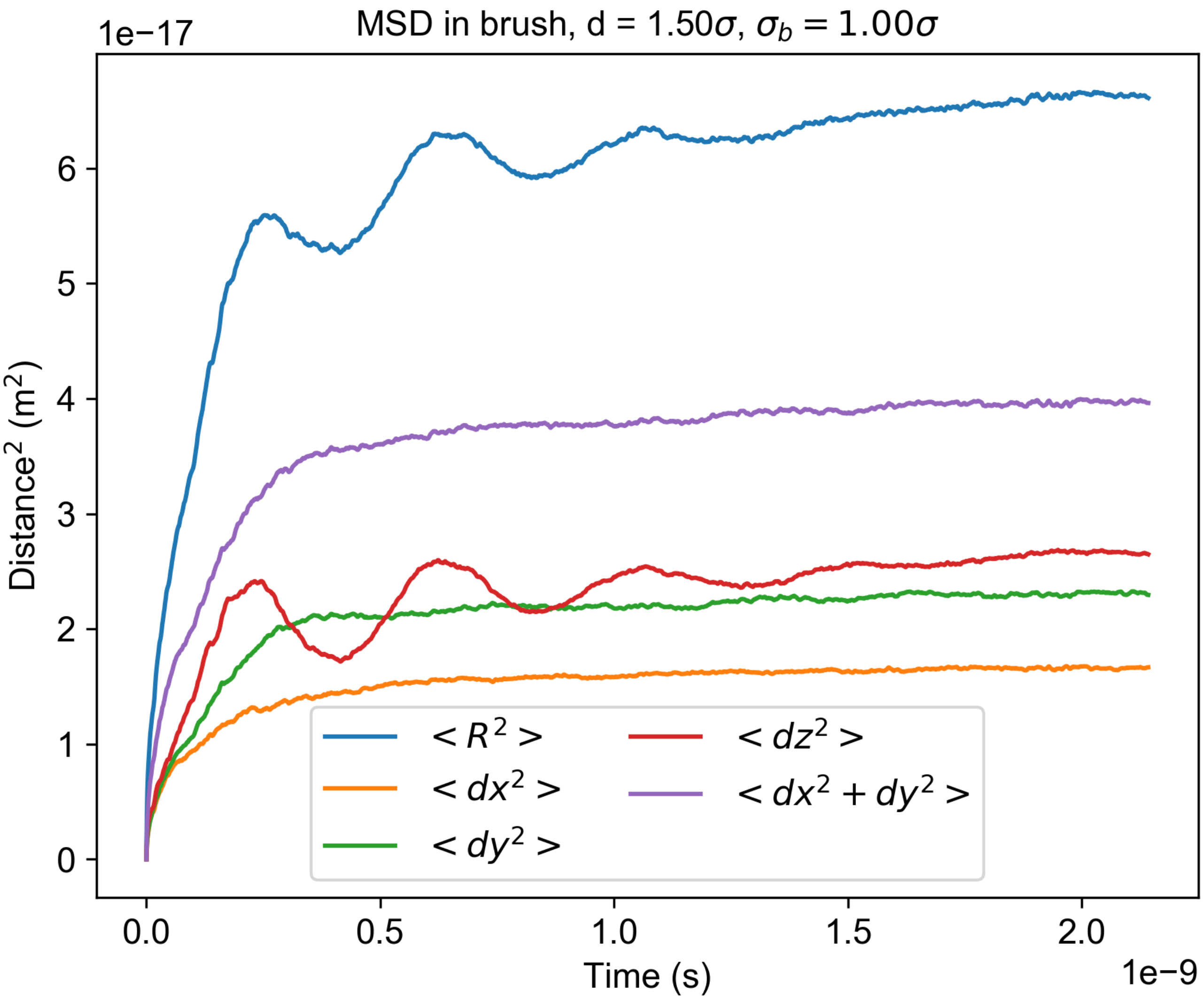

Figure S3. The mean squared displacement of the free particle for $d = 1.5\sigma$. Entrapment of the free particle causes convergence of the mean square displacement for $d = 1.5\sigma$.

Table S2. p_{next} for the custom models. All models are made by changing the probability p_{next} from the approach of (Mackie and Meares, 1955). Custom model 8 is the one given in the paper.

Model	p_{next}	Model	p_{next}	Model	p_{next}
1	$(1-k)\phi$	4	$k(1-\phi)^f$	7	$k(1-\phi)^{-1/d}$
2	$k(1-\phi)$	5	$k+(k-1)(1-\phi)^f$	8	$k+(k-1)(1-\phi)$
3	k	6	$k(1-\phi)^d$	9	$k+(1-k)(1-\phi)$

Table S3. The power laws. The first power law is the one used in the paper.

Model	Power law	Power law 2
D/D_{bulk}	$1-d^n k$	$1-d^n$

Mackie, J. S. and Meares, P. (1955). The Diffusion of Electrolytes in a Cation-Exchange Resin Membrane. I. Theoretical. *Proceedings of the Royal Society of London. Series A, Mathematical and Physical*

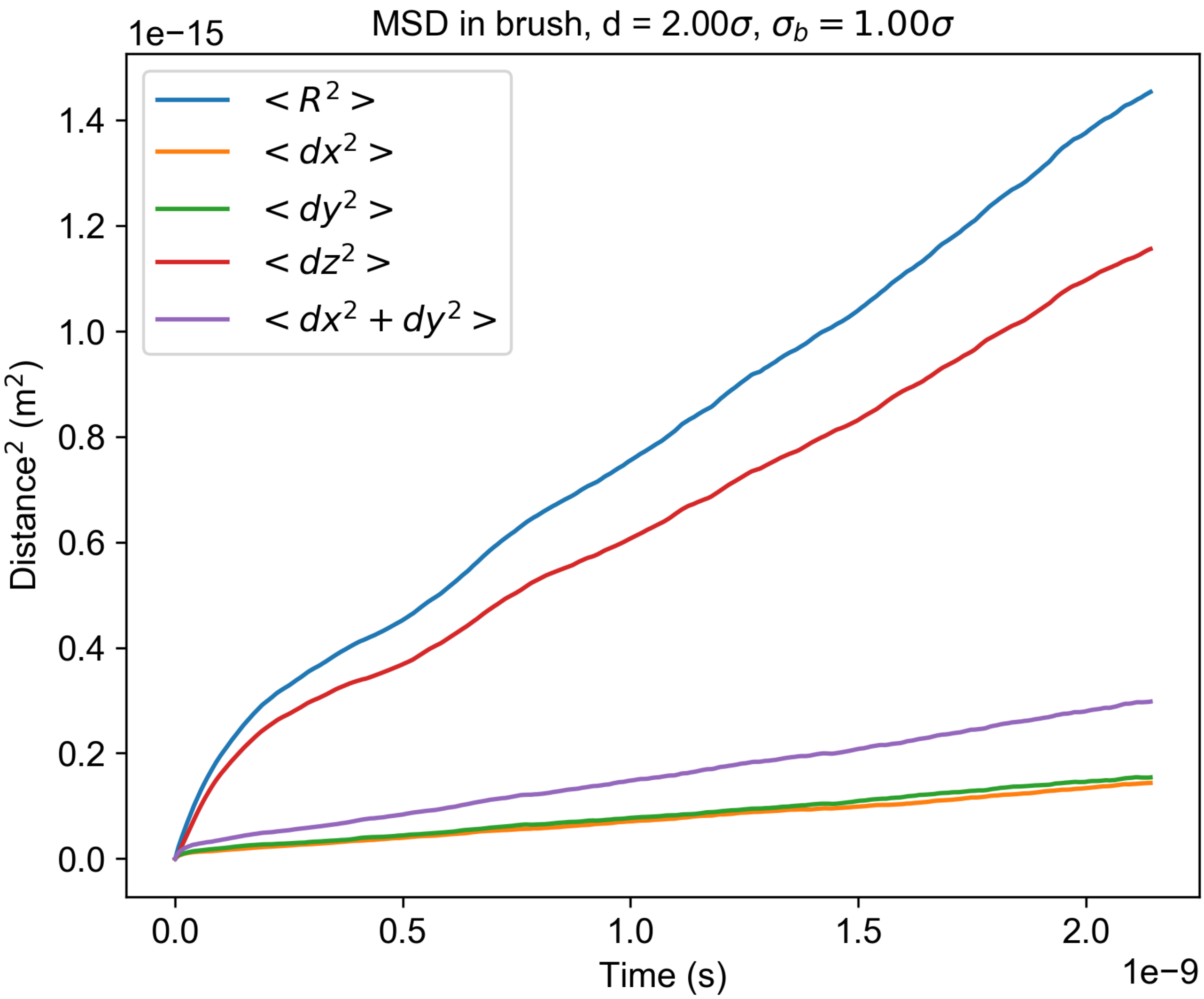

Figure S4. The mean squared displacement of the free particle for $d = 2\sigma$. The mean square displacement increases as a function of time for $d = 2\sigma$, indicating a reduced tendency for entrapment.

Sciences 232, 498–509

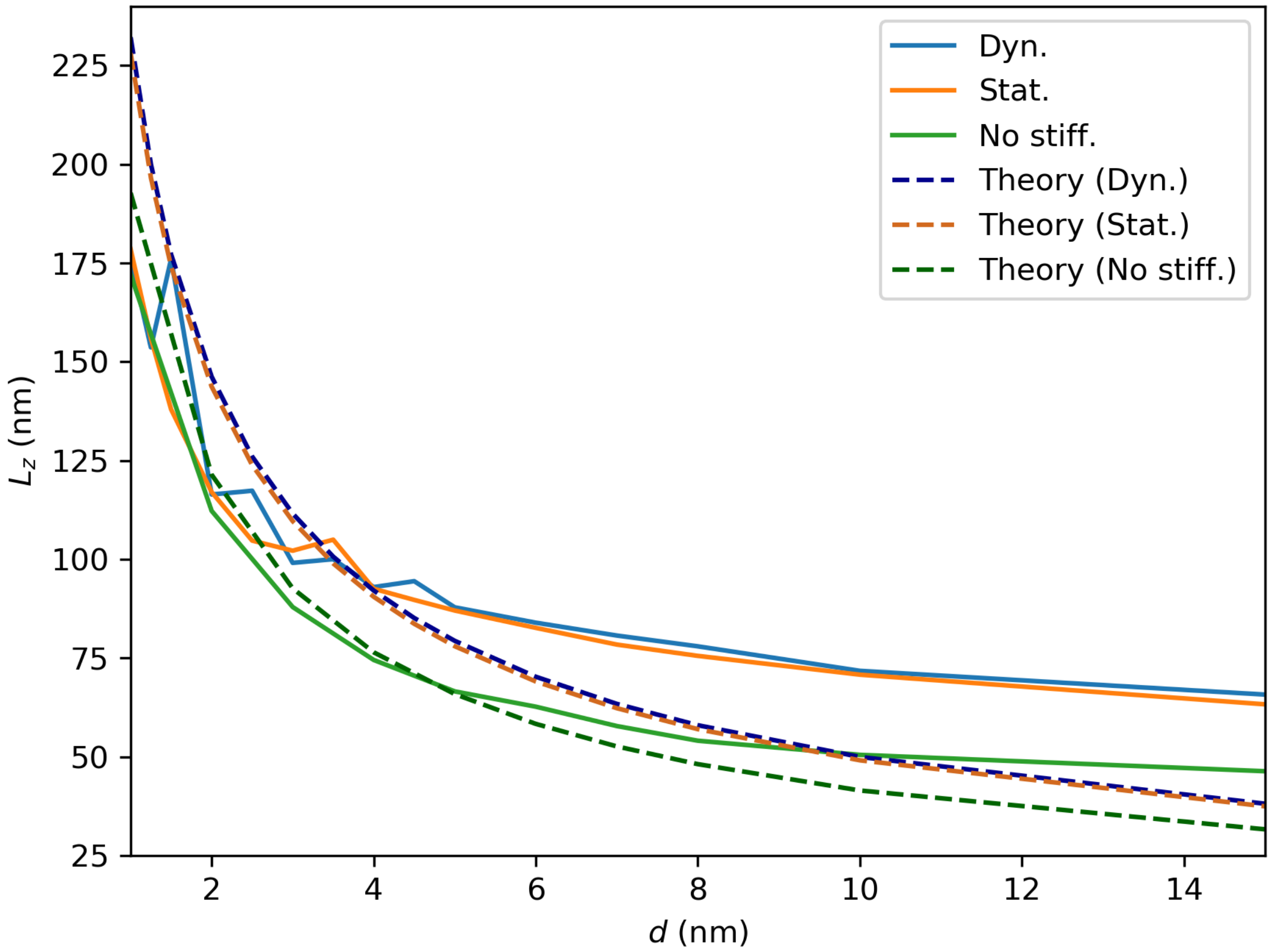

Figure S5. The average brush height L_z compared to theory for dynamic brushes, static brushes and brushes with no bending stiffness. The theory curves are fits to the expected scaling $L_z = Ad^{-2/3}$, which is stated in for instance (Attili et al., 2012).

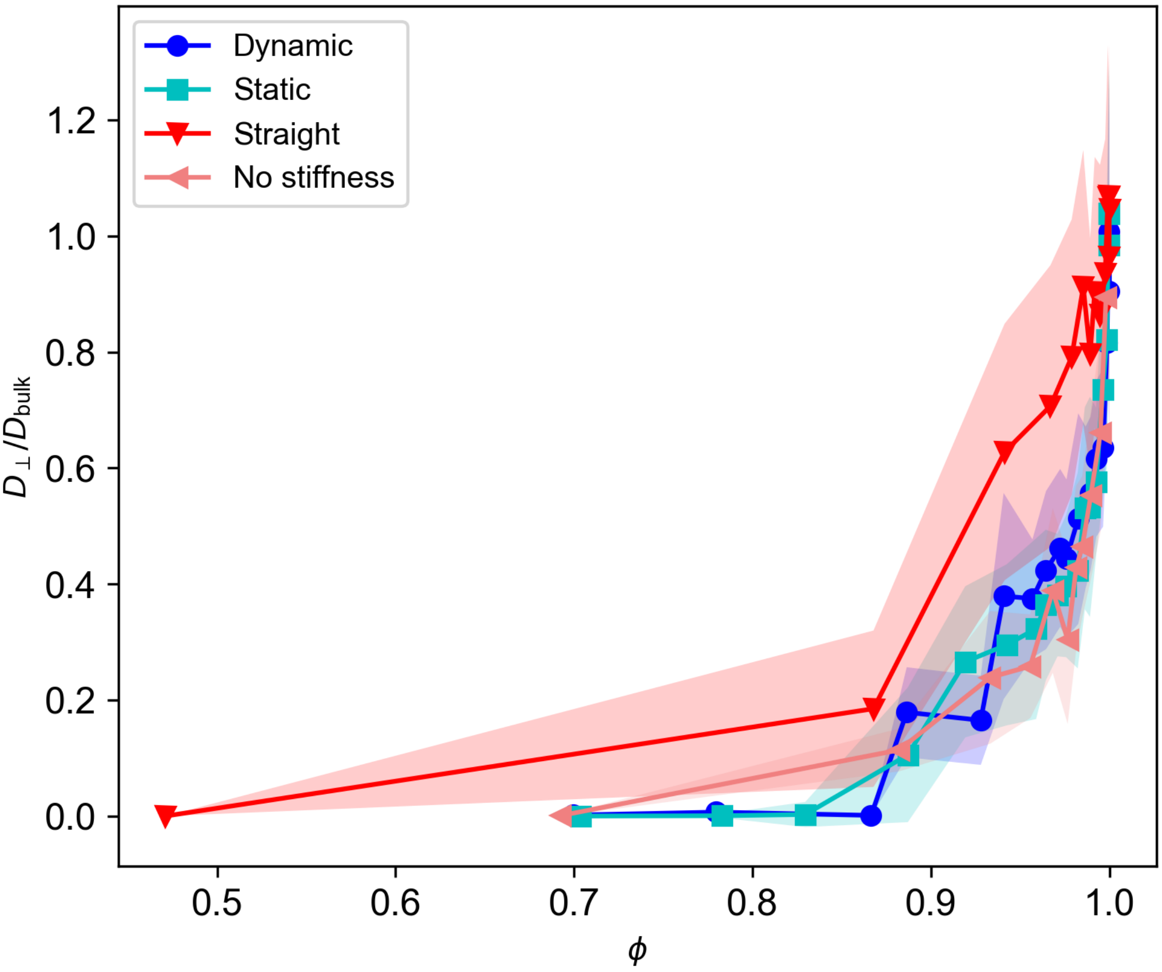

Figure S6. $D_\perp/D_\text{bulk}$ vs ϕ for $d = 5\sigma$. The diffusion constants for systems with dynamic chains, static chains and chains without stiffness agree well within the standard deviation. The system of straight, immobile chains exhibit a higher diffusion constant than the rest.

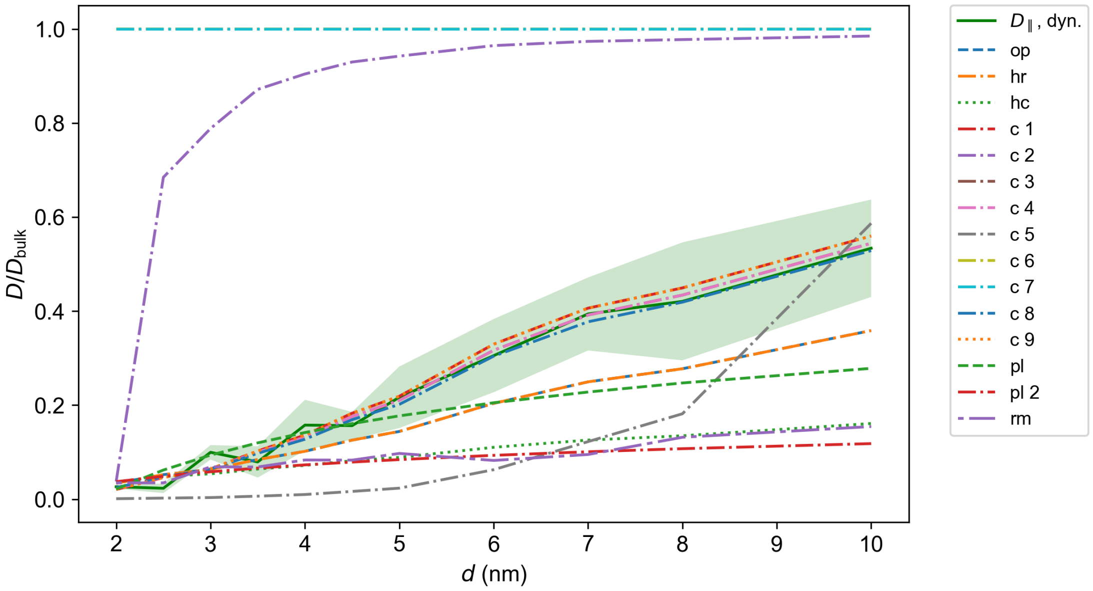

Figure S7. All the fits to $D_{\parallel}/D_{\mathrm{bulk}}$ for the dynamic brush. op - Ordered packings; hr - hyperbola of revolution; hc - heterogeneous catalyst; c - custom model; pl - power law; rm - cation-exchange resin membrane.

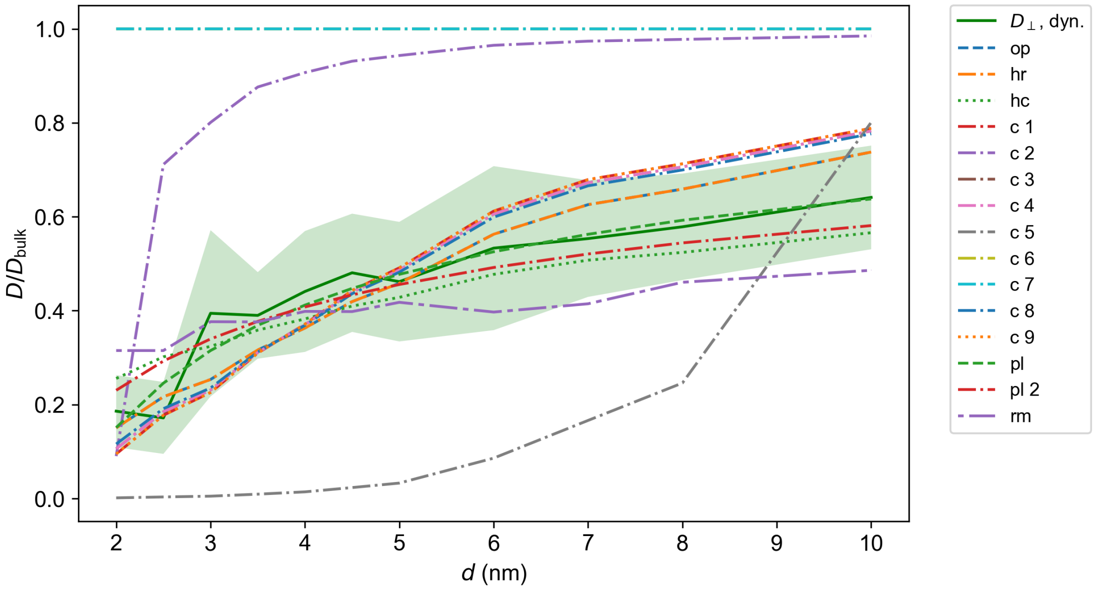

Figure S8. All the fits to $D_{\perp}/D_{\mathrm{bulk}}$ for the dynamic brush. op - Ordered packings; hr - hyperbola of revolution; hc - heterogeneous catalyst; c - custom model; pl - power law; rm - cation-exchange resin membrane.

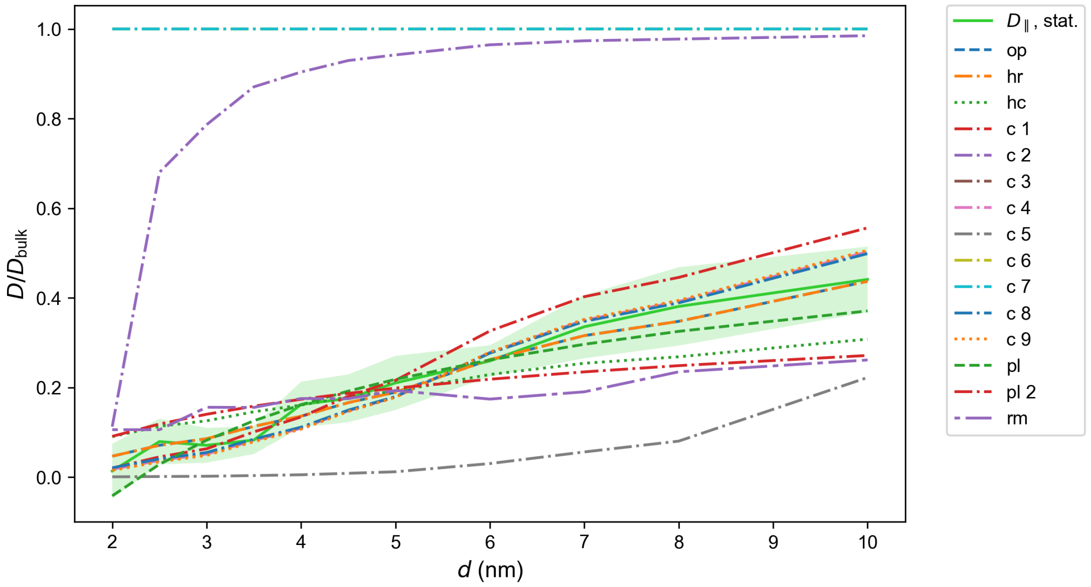

Figure S9. All the fits to $D_{\parallel}/D_{\mathrm{bulk}}$ for the static brush. op - Ordered packings; hr - hyperbola of revolution; hc - heterogeneous catalyst; c - custom model; pl - power law; rm - cation-exchange resin membrane.

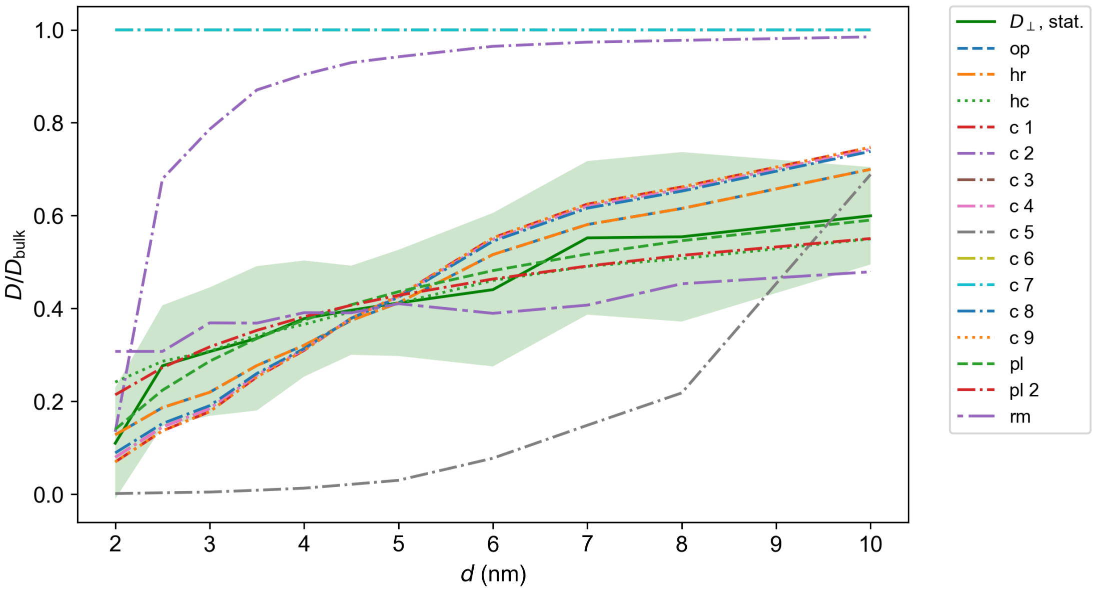

Figure S10. All the fits to $D_{\perp}/D_{\mathrm{bulk}}$ for the static brush. op - Ordered packings; hr - hyperbola of revolution; hc - heterogeneous catalyst; c - custom model; pl - power law; rm - cation-exchange resin membrane.

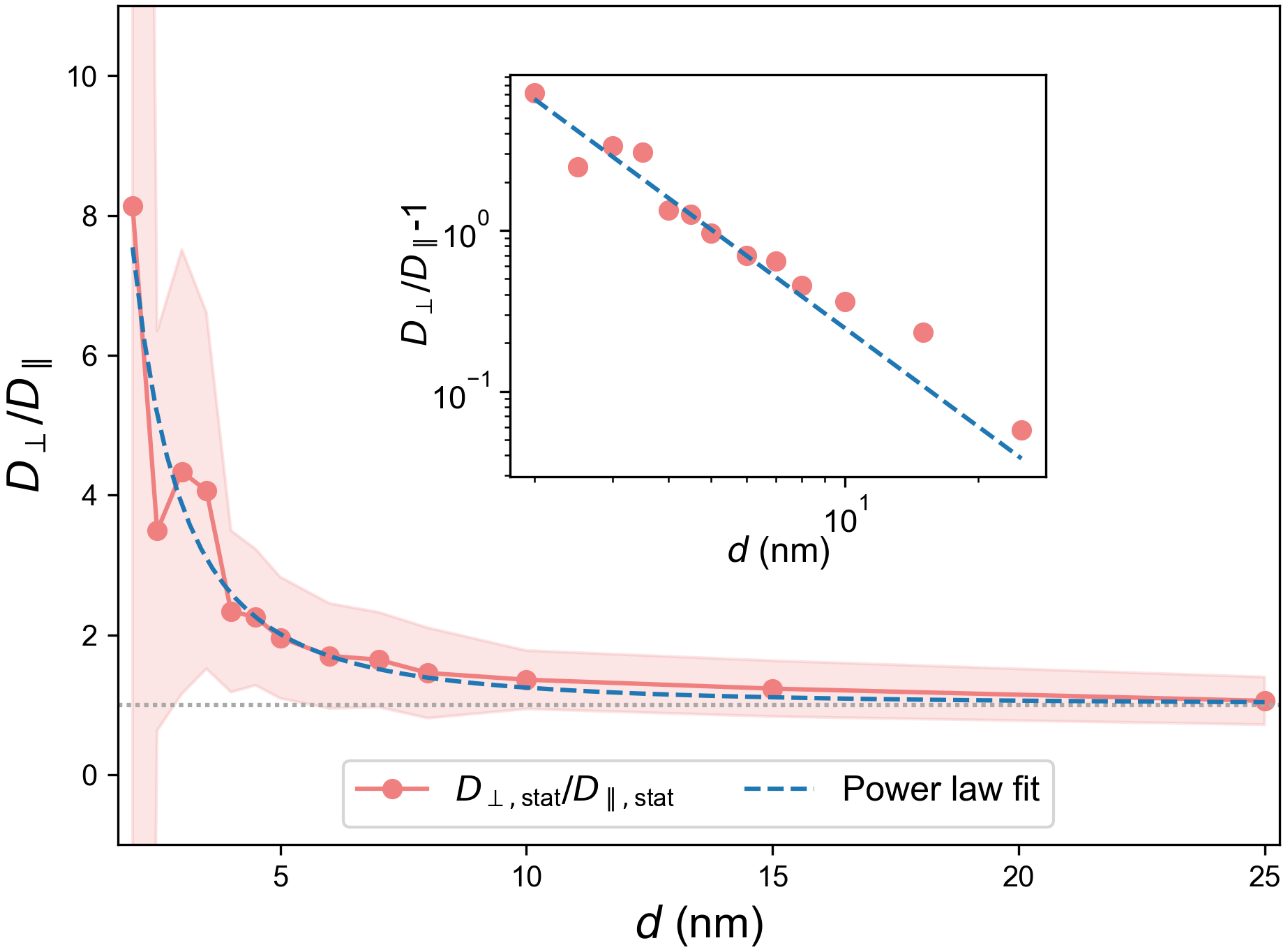

Figure S11. $D_\perp/D_\parallel$ vs d for the static brush, together with a fit to the power law $f(d) = Ad^{-l} + 1$. The fit is performed on in the range $d \in [2\sigma, 25\sigma]$. The standard deviation is indicated by shaded regions. Insert: A log-log plot of $D_\perp/D_\parallel - 1$ and Ad^{-l}.

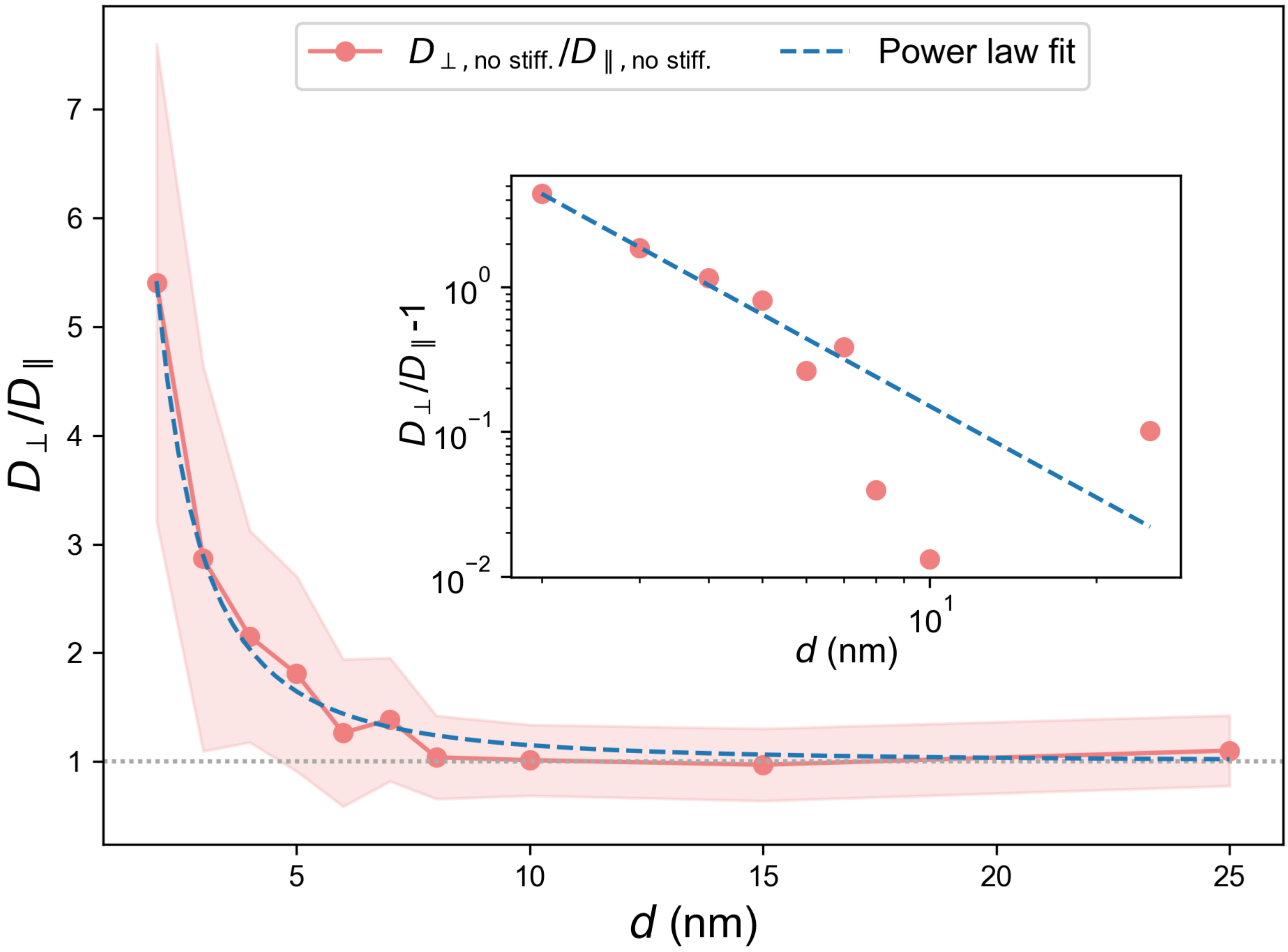

Figure S12. $D_\perp/D_\parallel$ vs d for the brush without a bending term, together with a fit to the power law $f(d) = Ad^{-l} + 1$. The fit is performed on in the range $d \in [2\sigma, 25\sigma]$. The standard deviation is indicated by shaded regions. Insert: A log-log plot of $D_\perp/D_\parallel - 1$ and Ad^{-l}. Note that $D_\perp/D_\parallel - 1$ fell below zero and is therefore not visible in the log-log plot.

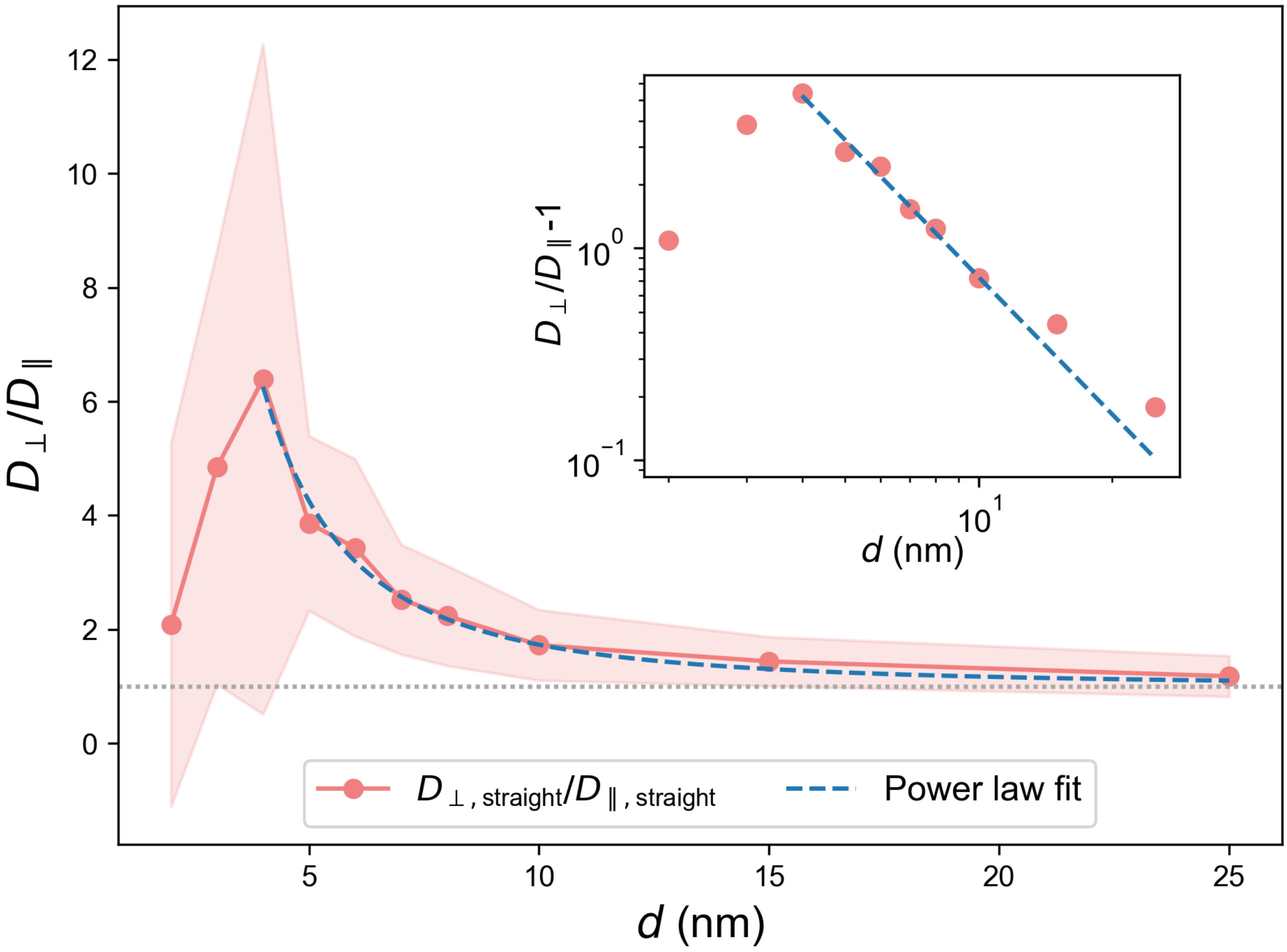

Figure S13. $D_\perp/D_\parallel$ vs d for the straight system, together with a fit to the power law $f(d) = Ad^{-l} + 1$. The fit is performed on in the range $d \in [4\sigma, 25\sigma]$. The standard deviation is indicated by shaded regions. Insert: A log-log plot of $D_\perp/D_\parallel - 1$ and Ad^{-l}.

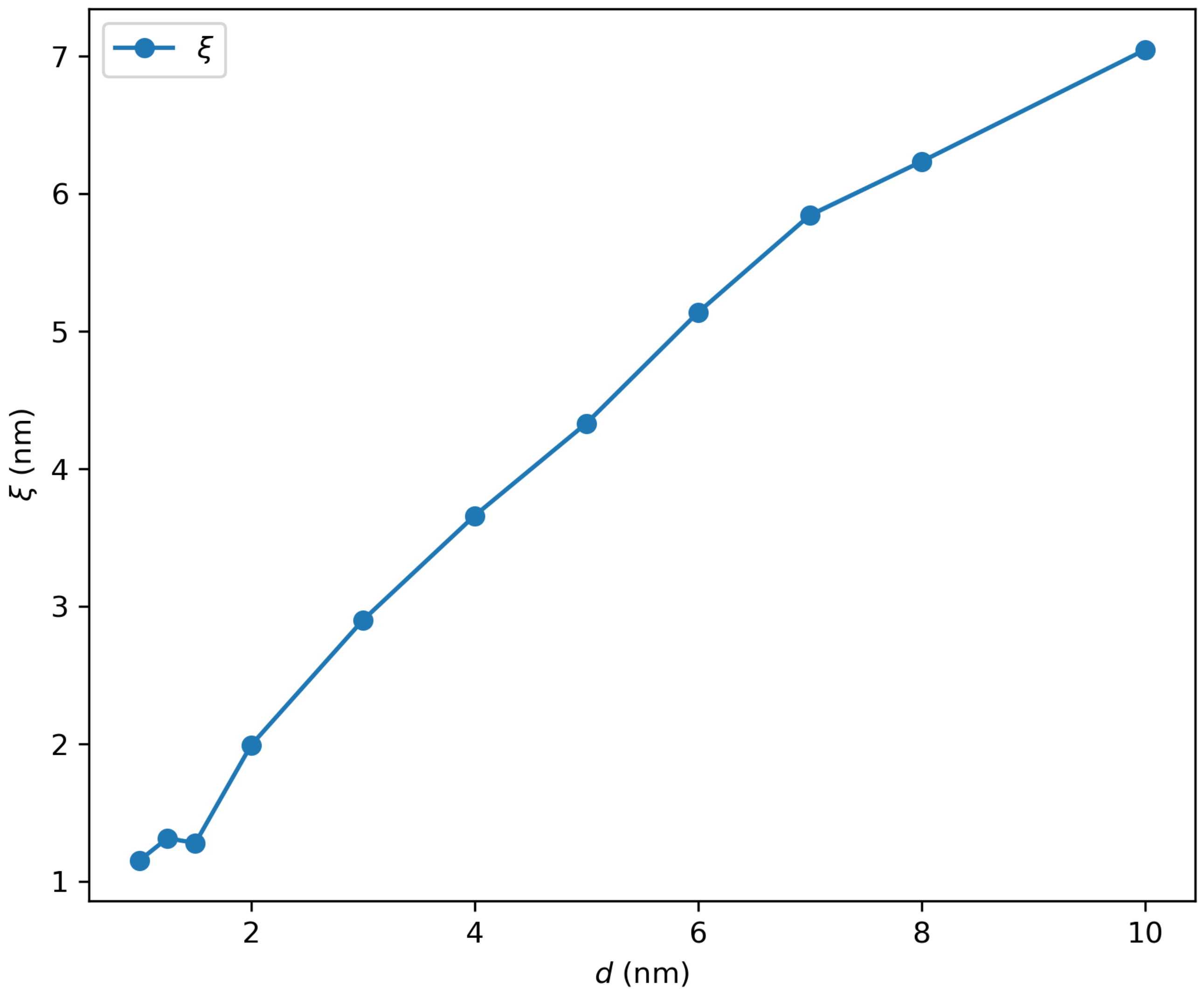

Figure S14. Correlation length ξ vs d for the dynamic system. The correlation length is the average distance between a bead on one chain and the closest bead on another chain.
